# Supplementary material for: Development of on-farm AMF inoculum production for sustainable agriculture in Senegal
Source: PLoS One. 2024 Nov 27;19(11):e0310065. doi: 10.1371/journal.pone.0310065 (PMC11602082; doi:10.1371/journal.pone.0310065)
Supplement: S1 Table — aThe mean data was obtained on sample composed of equal inoculum aliquots of 6 repetitions. Arbuscular mycorrhizal fungi root colonization rate was assessed using the method outlined by Trouvelot et al. [36]. (DOCX) [file pone.0310065.s001.docx]

| Substrate | Substrate proportion | Inoculation | Mean^a^ mycorhization intensity (%) | Mean mycorhization frequencies (%) | Mean Number spores (100-1g of substrate) |
| --- | --- | --- | --- | --- | --- |
| peanut shell | substrate | Inoc | 53.9 | 90 | 712 |
| peanut shell | substrate | Control | 0 | 0 | 0 |
| peanut shell | substrate/sand (50/50) | Inoc | 29.2 | 55 | 639 |
| peanut shell | substrate/sand (50/50) | Control | 0 | 0 | 0 |
| rice husk | substrate | Inoc | 29.7 | 62 | 543 |
| rice husk | substrate | Control | 0 | 0 | 0 |
| rice husk | substrate/sand (50/50) | Inoc | 30.5 | 78 | 537 |
| rice husk | substrate/sand (50/50) | Control | 0 | 0 | 0 |
| rice husk | substrate | Inoc | 16.9 | 44 | 271 |
| rice husk | substrate | Control | 0 | 0 | 0 |
| rice husk | substrate/sand (50/50) | Inoc | 15.8 | 51 | 101 |
| bagasse | substrate/sand (50/50) | Control | 0 | 0 | 0 |
| bagasse | substrate | Inoc | 7.1 | 25 | 18 |
| bagasse | substrate | Control | 0 | 0 | 0 |
| bagasse | substrate/sand (50/50) | Inoc | 4.3 | 7 | 21 |
| bagasse | substrate/sand (50/50) | Control | 0 | 0 | 0 |
| sand | substrate | Inoc | 54.4 | 64 | 681 |
| sand | substrate | Control | 0 | 0 | 0 |
| sand | substrate/sand (50/50) | Inoc | 29.2 | 55 | 591 |
| sand | substrate/sand (50/50) | Control | 0 | 0 | 0 |

**S2 Table.** Characteristics of the inoculum produced on agricultural residues alone or mixed with sand in Leonard jars

^a^The mean data was obtained on sample composed of equal inoculum aliquots of 6 repetitions. Arbuscular mycorrhizal fungi root colonization rate was assessed using the method outlined by Trouvelot et al. [36].

**S2 Table.** ANOVA of the mycorrhization parameters obtained on agricultural residues in Leonard jars

| **Mycorrhization**  **intensity** | Sum Sq | Mean Sq | F value | Pr(>F) |  |
| --- | --- | --- | --- | --- | --- |
| Substrate | 1007 | 252 | 2.191 | 0.127 |  |
| Inoculation | 3672 | 3672 | 31.944 | 7.9e-05 | *** |
| Substrate | 140 | 140 | 1.222 | 0.289 |  |
| Residuals | 1494 | 115 |  |  |  |
| **Mycorrhization frequencies** |  |  |  |  |  |
| Substrate | 2111 | 528 | 2.285 | 0.116 |  |
| Inoculation | 14098 | 14098 | 61.048 | 2.89e-06 | *** |
| Substrate | 76 | 76 | 0.329 | 0.576 |  |
| Residuals | 3002 | 231 |  |  |  |
| **Number of spores** |  |  |  |  |  |
| Substrat | 341123 | 85281 | 3.108 | 0.0533 | . |
| Inoculation | 846250 | 846250 | 30.845 | 9.32e-05 | *** |
| Substrate | 5645 | 5645 | 0.206 | 0.6576 |  |
| Residuals | 356665 | 27436 |  |  |  |

***. statistical significance of treatments at P-value < 0.05. The mean data was obtained on sample composed

of equal inoculum aliquots of 6 repetitions.
