# Supplementary material for: Development of on-farm AMF inoculum production for sustainable agriculture in Senegal
Source: PLoS One. 2024 Nov 27;19(11):e0310065. doi: 10.1371/journal.pone.0310065 (PMC11602082; doi:10.1371/journal.pone.0310065)
Supplement: S2 Table — aThe mean data was obtained on sample composed of equal inoculum aliquots of 4 repetitions. Arbuscular mycorrhizal fungi root colonization rate was assessed using the method outlined by Trouvelot et al. [36]. (DOCX) [file pone.0310065.s002.docx]

**S3 Table.** Characteristics of the inoculum produced on agricultural residues in pots

| Substrate | Inoculation | Mean^a^ mycorhization intensity (%) | Mean mycorhization frequencies (%) | Mean Number spores (100^-1^g of substrate) |
| --- | --- | --- | --- | --- |
| peanut shell | Inoc | 46.3 | 86 | 527 |
| peanut shell | Control | 0 | 0 | 0 |
| rice husk | Inoc | 37.4 | 84 | 503 |
| rice husk | Control | 0 | 0 | 0 |
| ears of millet | Inoc | 20.48 | 31 | 278 |
| ears of millet | Control | 0 | 0 | 0 |
| bagasse | Inoc | 6.2 | 19 | 27 |
| bagasse | Control | 0 | 0 | 0 |
| sand | Inoc | 41.7 | 68 | 548 |
| sand | Control | 0 | 0 | 0 |

^a^The mean data was obtained on sample composed of equal inoculum aliquots of 4 repetitions. Arbuscular mycorrhizal fungi root

colonization rate was assessed using the method outlined by Trouvelot et al. [36].

**S3 Table.** ANOVA analysis of substrate type and inoculation effects on the mycorrhization parameters

obtained on corn plants in pots

| **Mycorrhization intensity** | Sum Sq | Mean Sq | F value | Pr(>F) |  |
| --- | --- | --- | --- | --- | --- |
| Substrate | 556.8 | 139.2 | 1.00 | 0.5000 |  |
| Inoculation | 2312.8 | 2312.8 | 16.62 | 0.0151 | * |
| Residuals | 556.8 | 139.2 |  |  |  |
| **Mycorrhization frequencies** |  |  |  |  |  |
| Substrate | 1905 | 476 | 1.00 | 0.500 |  |
| Inoculation | 8294 | 8294 | 17.42 | 0.014 | * |
| Residuals | 1905 | 476 |  |  |  |
| **Number of spores** |  |  |  |  |  |
| Substrate | 99959 | 24990 | 1.00 | 0.5000 |  |
| Inoculation | 354569 | 354569 | 14.19 | 0.0197 | * |
| Residuals | 99959 | 24990 |  |  |  |

*, statistical significance of treatments or interactions at p-value < 0.05. The mean data was obtained on sample

composed of equal inoculum aliquots of 4 repetitions.
