# Supplementary material for: Development of on-farm AMF inoculum production for sustainable agriculture in Senegal
Source: PLoS One. 2024 Nov 27;19(11):e0310065. doi: 10.1371/journal.pone.0310065 (PMC11602082; doi:10.1371/journal.pone.0310065)
Supplement: S3 Table — (DOCX) [file pone.0310065.s003.docx]

**S4 Table.** Characteristics of the inoculum produced on the peanut shell at the pilot site (ANOVA with R, 2023)

| **Traitement** | **Repetition** | **Spores** | **Frequence** | **Intensite** |
| --- | --- | --- | --- | --- |
| control | R1 | 9 | 38.33 | 2.3 |
| control | R2 | 17 | 36.67 | 0.5 |
| control | R3 | 0 | 0.00 | 0.00 |
| control | R4 | 0 | 0.00 | 0.00 |
| control | R5 | 5 | 0.00 | 0.00 |
| *Glomus aggregatum* | R1 | 20 | 91.67 | 8.3 |
| *Glomus aggregatum* | R2 | 5 | 96.67 | 21.2 |
| *Glomus aggregatum* | R3 | 15 | 85.00 | 21.3 |
| *Glomus aggregatum* | R4 | 12 | 83.33 | 24 |
| *Glomus aggregatum* | R5 | 15 | 88.00 | 24 |
| *Glomus fasciculatum* | R1 | 10 | 66.67 | 15 |
| *Glomus fasciculatum* | R2 | 25 | 43.33 | 12.3 |
| *Glomus fasciculatum* | R3 | 4 | 80.00 | 22.5 |
| *Glomus fasciculatum* | R4 | 34 | 86.67 | 12.5 |
| *Glomus fasciculatum* | R5 | 4 | 75.33 | 6 |
| *Glomus mosseae* | R1 | 13 | 78.33 | 10.2 |
| *Glomus mosseae* | R2 | 13 | 45.00 | 6.3 |
| *Glomus mosseae* | R3 | 15 | 85.00 | 12.2 |
| *Glomus mosseae* | R4 | 15 | 50.00 | 9.3 |
| *Glomus mosseae* | R5 | 12 | 52.00 | 5.3 |
| *Rhizophagus irregularis* | R1 | 17 | 68.33 | 5.3 |
| *Rhizophagus irregularis* | R2 | 36 | 41.67 | 6 |
| *Rhizophagus irregularis* | R3 | 38 | 20.00 | 9.3 |
| *Rhizophagus irregularis* | R4 | 5 | 41.67 | 15 |
| *Rhizophagus irregularis* | R5 | 10 | 39.33 | 12.3 |

| summary(model1) spores |  |  |
| --- | --- | --- |
| Df Sum Sq Mean Sq F value Pr(>F) | | |
| Traitement 4 575.8 143.94 1.259 0.327 | | |
| > summary(model2)intensity |  |  |
| Df Sum Sq Mean Sq F value Pr(>F) | | |
| Traitement 4 959.7 239.92 12.592 8.07e-05 *** | | |
| summary(model3)FREQUENCE |  |  |
| Df Sum Sq Mean Sq F value Pr(>F) | | |
| Traitement 4 15999 4000 14.63 3.28e-05 *** | | |

|  |  | *G. agregatum* IR27 | *G. fasciculatum* | *F. mosseae* | *R. irregularis* | Control |
| --- | --- | --- | --- | --- | --- | --- |
| Mean Number  of spores (100^-1^g substrate) | Average | **13.4 a** | **15.4 a** | **13.6 a** | **21.2 a** | **6.2 a** |
|  | Std | 5.5 | 13.48 | 1.34 | 15.06 | 7.12 |
|  | Repeat | 5 | 5 | 5 | 5 | 5 |
|  | Min | 5 | 4 | 12 | 5 | 0 |
|  | Max | 20 | 34 | 15 | 38 | 17 |
| Mean Mycorrhization intensity (%) | Average | **19.76 a** | **13.66 ab** | **8.66 bc** | **9.58 b** | **0.91 c** |
|  | Std | 6.55 | 5.95 | 2.84 | 4.12 | 0.81 |
|  | Repeat | 5 | 5 | 5 | 5 | 5 |
|  | Min | 8.3 | 6 | 5.3 | 5.3 | 0.2 |
|  | Max | 24 | 22.5 | 12.2 | 15 | 2.1 |
| Mean Mycorrhization frequency (%) | Average | **88.93 a** | **70.4 a** | **62.07 ab** | **42.20 bc** | **15.0 c** |
|  | Std | 5.36 | 16.79 | 18.23 | 17.21 | 20.55 |
|  | Repeat | 5 | 5 | 5 | 5 | 5 |
|  | Min | 83.33 | 43.33 | 45 | 20 | 0 |
|  | Max | 96.67 | 86.67 | 85 | 68.33 | 38.33 |
