# Supplementary material for: Development of on-farm AMF inoculum production for sustainable agriculture in Senegal
Source: PLoS One. 2024 Nov 27;19(11):e0310065. doi: 10.1371/journal.pone.0310065 (PMC11602082; doi:10.1371/journal.pone.0310065)
Supplement: S1 Fig — (DOCX) [file pone.0310065.s004.docx]

**S2 Fig.** Effects of the treatments on the mycorhization using as variables the number of spores, mycorrhization intensity and frequency (Box Plot with R. 2023)

| **Water** | **Substrate** | **Treatment** | **Repetition** | **Spores** | **Frequency** | **Intensity** |
| --- | --- | --- | --- | --- | --- | --- |
| Salty | Sterile | LCM | R1 | 104 | 0 | 0 |
| Salty | Sterile | LCM | R2 | 106 | 0 | 0 |
| Salty | Sterile | LCM | R3 | 101 | 0 | 0 |
| No_Salty | Sterile | LCM | R1 | 136 | 33.66 | 7.99 |
| No_Salty | Sterile | LCM | R2 | 206 | 36.11 | 9 |
| No_Salty | Sterile | LCM | R3 | 292 | 30.22 | 5 |
| No_Salty | No_Sterile | LCM | R1 | 79 | 5 | 0.02 |
| No_Salty | No_Sterile | LCM | R2 | 92 | 3 | 0.01 |
| No_Salty | No_Sterile | LCM | R3 | 66 | 7 | 0.03 |
| Salty | No_Sterile | LCM | R1 | 21 | 1.67 | 0.19 |
| Salty | No_Sterile | LCM | R2 | 47 | 1.7 | 0.15 |
| Salty | No_Sterile | LCM | R3 | 32 | 1.65 | 0.18 |
| Salty | Sterile | T | R1 | 0 | 0 | 0 |
| Salty | Sterile | T | R2 | 3 | 0 | 0 |
| Salty | Sterile | T | R3 | 5 | 0 | 0 |
| No_Salty | No_Sterile | T | R1 | 20 | 1.83 | 1 |
| No_Salty | No_Sterile | T | R2 | 33 | 1.63 | 0 |
| No_Salty | No_Sterile | T | R3 | 41 | 1.56 | 2 |
| No_Salty | Sterile | T | R1 | 0 | 0 | 0 |
| No_Salty | Sterile | T | R2 | 3 | 0 | 0 |
| No_Salty | Sterile | T | R3 | 4 | 0 | 0 |
| Salty | No_Sterile | T | R1 | 10 | 0 | 0 |
| Salty | No_Sterile | T | R2 | 11 | 0 | 0 |
| Salty | No_Sterile | T | R3 | 15 | 0 | 0 |
