# Supplementary material for: Development of on-farm AMF inoculum production for sustainable agriculture in Senegal
Source: PLoS One. 2024 Nov 27;19(11):e0310065. doi: 10.1371/journal.pone.0310065 (PMC11602082; doi:10.1371/journal.pone.0310065)
Supplement: S2 Fig — mycorrhization intensity and frequency obtained on corn plants (ANOVA with R. 2023). (DOCX) [file pone.0310065.s005.docx]

**S3 Fig.** Type III sum squares analysis of the treatment effects on the mycorhization using as variables spore number. mycorrhization intensity and frequency obtained on corn plants (ANOVA with R. 2023)

| Parameters |  | ddl | Sum of squares of deviations | Mean square | F Value | Pr(>F) |
| --- | --- | --- | --- | --- | --- | --- |
| Spores | Substrate | 1 | 10127 | 10127 | 13.032 | 0.002842** |
|  | Water | 1 | 11137 | 11137 | 14.332 | 0.002007** |
|  | Treatments | 1 | 53865 | 53865 | 69.316 | 8.58e-07*** |
|  | Repetitions | 2 | 2283 | 1141 | 1.469 | 0.263625 |
|  | Substrate:Water | 1 | 672 | 672 | 0.865 | 0.368156 |
|  | Substrate:Treatments | 1 | 21780 | 21780 | 28.028 | 0.000113 *** |
|  | Water:Treatments | 1 | 6767 | 6767 | 8.708 | 0.010526 * |
|  | Substrate:Water:Treatments | 1 | 2501 | 2501 | 3.218 | 0.094432 |
|  | Residuals | 14 | 14 10879 | 777 |  |  |
| Intensity | Substrate | 1 | 14.122 | 14.122 | 19.137 | 0.000635 *** |
|  | Water | 1 | 25.072 | 25.072 | 33.976 | 4.38e-05 *** |
|  | Treatments | 1 | 15.958 | 15.958 | 21.625 | 0.000375 *** |
|  | Repetitions | 2 | 0.324 | 0.324 | 0.219 | 0.805865 |
|  | Substrate:Water | 1 | 15.763 | 15.763 | 21.361 | 0.000396 *** |
|  | Substrate:Treatments | 1 | 24.827 | 24.827 | 21.361 | 0.000396 *** |
|  | Water:Treatments | 1 | 14.307 | 14.307 | 19.388 | 0.000601 *** |
|  | Substrate:Water:Treatments | 1 | 26.988 | 26.988 | 19.388 | 0.000601 *** |
|  | Residuals | 14 | 10.331 | 0.738 |  |  |
| Frequency | Substrate | 1 | 234.1 | 234.1 | 129.759 | 1.82e-08 *** |
|  | Water | 1 | 550.9 | 550.9 | 305.431 | 6.64e-11 *** |
|  | Treatments | 1 | 550.9 | 550.9 | 305.431 | 6.64e-11 *** |
|  | Repetitions | 2 | 0.3 | 0.1 | 0.082 | 0.922 |
|  | Substrate:Water | 1 | 301.0 | 301.0 | 166.851 | 3.61e-09 *** |
|  | Substrate:Treatments | 1 | 301.0 | 301.0 | 166.851 | 3.61e-09 *** |
|  | Water:Treatments | 1 | 458.9 | 458.9 | 254.424 | 2.25e-10 *** |
|  | Substrate:Water:Treatments | 1 | 376.3 | 376.3 | 208.600 | 8.39e-10 *** |
|  | Residuals | 14 | 25.3 | 1.8 |  |  |

Frequency

Intensity

Frequency

Intensity
