# Supplementary material for: Development of on-farm AMF inoculum production for sustainable agriculture in Senegal
Source: PLoS One. 2024 Nov 27;19(11):e0310065. doi: 10.1371/journal.pone.0310065 (PMC11602082; doi:10.1371/journal.pone.0310065)
Supplement: S3 Fig — (DOCX) [file pone.0310065.s006.docx]

**S4 Fig.** Mean number of spores obtained by classical and improved extraction methods when using peanut shells as growth substrate (ANOVA with XLSTAT)

| **methods** | **treatment** | **Spores** |
| --- | --- | --- |
| classical | LCM | 116 |
| classical | LCM | 97 |
| classical | LCM | 105 |
| improved | LCM | 115 |
| improved | LCM | 130 |
| improved | LCM | 126 |
| classical | INO+salted Water | 15 |
| classical | INO+salted Water | 19 |
| classical | INO+salted Water | 20 |
| improved | INO+salted Water | 35 |
| improved | INO+salted Water | 27 |
| improved | INO+salted Water | 24 |
| classical | INO+unsalted water | 45 |
| classical | INO+unsalted water | 55 |
| classical | INO+unsalted water | 35 |
| improved | INO+unsalted water | 79 |
| improved | INO+unsalted water | 76 |
| improved | INO+unsalted water | 88 |
| classical | control | 3 |
| classical | control | 1 |
| classical | control | 5 |
| improved | control | 2 |
| improved | control | 2 |
| improved | control | 3 |

| Contrast | Difference | Standardized difference | Critical value | Pr > Diff | Significance |
| --- | --- | --- | --- | --- | --- |
| Control vs LCM | -112.167 | -30.977 | 2.110 | **< 0.0001** | Yes |
| Control vs INO+unsalted water | -60.333 | -16.662 | 2.110 | **< 0.0001** | Yes |
| Control vs INO+salted water | -20.667 | -5.708 | 2.110 | **< 0.0001** | Yes |
| INO+ salted water vs LCM | -91.500 | -25.270 | 2.110 | **< 0.0001** | Yes |
| INO+ salted water vs INO+unsalted water | -39.667 | -10.955 | 2.110 | **< 0.0001** | Yes |
| INO+ unsalted water vs LCM | -51.833 | -14.315 | 2.110 | **< 0.0001** | Yes |
| LSD-value : |  |  | 7.639 |  |  |
