# Supplementary material for: Development of on-farm AMF inoculum production for sustainable agriculture in Senegal
Source: PLoS One. 2024 Nov 27;19(11):e0310065. doi: 10.1371/journal.pone.0310065 (PMC11602082; doi:10.1371/journal.pone.0310065)
Supplement: Attachment — Submitted filename: PONE-D-24-13955_review.pdf [file pone.0310065.s007.pdf]

PLOS ONE

Development of on-farm AMF inoculum production for sustainable agriculture in Senegal

--Manuscript Draft--

**Manuscript Number:**	PONE-D-24-13955
**Article Type:**	Research Article
**Full Title:**	Development of on-farm AMF inoculum production for sustainable agriculture in Senegal
**Short Title:**	On-farm AMF inoculum
**Corresponding Author:**	Tatiana Krasova Wade, Ph.D<br>Institut de recherche pour le developpement<br>Dakar, SENEGAL
**Keywords:**	Arbuscular mycorrhizal fungi (AMF); Inoculum production; Agricultural residual products
**Abstract:**	The integration of endomycorrhizal fungi into agricultural practices as inoculum offers the potential to enhance plant productivity while reducing the reliance on costly chemical fertilizers, which are not only economically onerous but also environmentally detrimental. Mycorrhizal fungi play a crucial role in facilitating plant access to essential mineral elements (such as Phosphorus, Potassium, etc.) and water, particularly in soils characterized by arid and semi-arid conditions where these resources are often limited. Despite the evident benefits, the production of arbuscular mycorrhizal fungi (AMF) inoculum in Africa remains constrained by technological limitations, with current operations typically confined to small or medium-scale endeavors. This research aims to address this limitation by exploring the feasibility of producing mycorrhizal inoculum on a semi-industrial farm scale, achieved through the control and stabilization of production parameters. Laboratory experiments were conducted to evaluate the multiplication of mycorrhizal fungi, including Glomus aggregatum IR27, Funneliformis mosseae, Rhizophagus irregularis, and Glomus fasciculatum R10. Various agricultural residues, such as peanut shells, rice husks, sugar cane bagasse and millet cobs, were tested as alternatives to conventional sand production supports. Significant results indicated that peanut shells can serve as a suitable substrate for upscaling and decentralizing production. Moreover, the study emphasized the critical considerations of irrigation water salt content and substrate sterilization as pivotal parameters to ensure optimal development of mycorrhizal propagules. This experimental endeavor serves as a pioneering initiative, laying the groundwork for the establishment of a local sector dedicated to the production and distribution of endomycorrhizal fungi inoculum in Senegal.
**Order of Authors:**	Christian Valentin Nadieline
	Antoine Le Quéré
	Cheikh Ndiaye
	Amadou Abib Diène
	Francis Do Rego
	Oumar Sadio
	Yoro Idrissa Thioye
	Marc Neyra
	Cheikh Mouhamed Fadel Kébé
	Tatiana Krasova Wade, Ph.D
**Additional Information:**	
**Question**	**Response**

Powered by Editorial Manager® and ProduXion Manager® from Aries Systems Corporation

**Financial Disclosure**<br><br>Enter a financial disclosure statement that describes the sources of funding for the work included in this submission. Review the submission guidelines for detailed requirements. View published research articles from *PLOS ONE* for specific examples.<br><br>This statement is required for submission and **will appear in the published article** if the submission is accepted. Please make sure it is accurate.<br><br>**Funded studies**<br>Enter a statement with the following details:<br>• Initials of the authors who received each award<br>• Grant numbers awarded to each author<br>• The full name of each funder<br>• URL of each funder website<br>• Did the sponsors or funders play any role in the study design, data collection and analysis, decision to publish, or preparation of the manuscript?<br><br>Did you receive funding for this work?	Yes
Please add funding details.<br>as follow-up to "**Financial Disclosure**<br><br>Enter a financial disclosure statement that describes the sources of funding for the work included in this submission. Review the submission guidelines for detailed requirements. View published research articles from *PLOS ONE* for specific examples.<br><br>This statement is required for submission and **will appear in the published article** if the submission is accepted. Please make sure it is accurate.	OAPI funding 27714 and IRD funding MySen

**Funded studies**<br>Enter a statement with the following details:<br>• Initials of the authors who received each award<br>• Grant numbers awarded to each author<br>• The full name of each funder<br>• URL of each funder website<br>• Did the sponsors or funders play any role in the study design, data collection and analysis, decision to publish, or preparation of the manuscript?<br><br>Did you receive funding for this work?"	
Please select the country of your main research funder (please select carefully as in some cases this is used in fee calculation).<br>as follow-up to "**Financial Disclosure**<br><br>Enter a financial disclosure statement that describes the sources of funding for the work included in this submission. Review the submission guidelines for detailed requirements. View published research articles from *PLOS ONE* for specific examples.<br><br>This statement is required for submission and **will appear in the published article** if the submission is accepted. Please make sure it is accurate.<br><br>**Funded studies**<br>Enter a statement with the following details:<br>• Initials of the authors who received each award<br>• Grant numbers awarded to each author<br>• The full name of each funder<br>• URL of each funder website<br>• Did the sponsors or funders play any role in the study design, data collection and analysis, decision to publish, or preparation of the manuscript?<br><br>Did you receive funding for this work?"	FRANCE - FR
**Competing Interests**	The authors have declared that no competing interests exist

Powered by Editorial Manager® and ProduXion Manager® from Aries Systems Corporation

Use the instructions below to enter a competing interest statement for this submission. On behalf of all authors, disclose any competing interests that could be perceived to bias this work—acknowledging all financial support and any other relevant financial or non-financial competing interests.<br><br>This statement is required for submission and **will appear in the published article** if the submission is accepted. Please make sure it is accurate and that any funding sources listed in your Funding Information later in the submission form are also declared in your Financial Disclosure statement.<br><br>View published research articles from *PLOS ONE* for specific examples.<br><br>**NO authors have competing interests**<br><br>Enter: *The authors have declared that no competing interests exist.*<br><br>**Authors with competing interests**<br><br>Enter competing interest details beginning with this statement:<br><br>*I have read the journal's policy and the authors of this manuscript have the following competing interests: [insert competing interests here]*<br><br>* typeset	
**Ethics Statement**<br><br>Enter an ethics statement for this submission. This statement is required if the study involved:<br><br>• Human participants<br>• Human specimens or tissue<br>• Vertebrate animals or cephalopods<br>• Vertebrate embryos or tissues	No

• Field research<br><br>Write "N/A" if the submission does not require an ethics statement.<br><br>General guidance is provided below. Consult the submission guidelines for detailed instructions. **Make sure that all information entered here is included in the Methods section of the manuscript.**	

**Format for specific study types**<br><br>**Human Subject Research (involving human participants and/or tissue)**<br>• Give the name of the institutional review board or ethics committee that approved the study<br>• Include the approval number and/or a statement indicating approval of this research<br>• Indicate the form of consent obtained (written/oral) or the reason that consent was not obtained (e.g. the data were analyzed anonymously)<br><br>**Animal Research (involving vertebrate animals, embryos or tissues)**<br>• Provide the name of the Institutional Animal Care and Use Committee (IACUC) or other relevant ethics board that reviewed the study protocol, and indicate whether they approved this research or granted a formal waiver of ethical approval<br>• Include an approval number if one was obtained<br>• If the study involved *non-human primates*, add *additional details* about animal welfare and steps taken to ameliorate suffering<br>• If anesthesia, euthanasia, or any kind of animal sacrifice is part of the study, include briefly which substances and/or methods were applied<br><br>**Field Research**<br><br>Include the following details if this study involves the collection of plant, animal, or other materials from a natural setting:<br>• Field permit number<br>• Name of the institution or relevant body that granted permission	
**Data Availability**<br><br>Authors are required to make all data underlying the findings described fully available, without restriction, and from the time of publication. PLOS allows rare exceptions to address legal and ethical concerns. See the PLOS Data Policy and FAQ for detailed information.	Yes - all data are fully available without restriction

A Data Availability Statement describing where the data can be found is required at submission. Your answers to this question constitute the Data Availability Statement and **will be published in the article**, if accepted.<br><br>**Important:** Stating 'data available on request from the author' is not sufficient. If your data are only available upon request, select 'No' for the first question and explain your exceptional situation in the text box.<br><br>Do the authors confirm that all data underlying the findings described in their manuscript are fully available without restriction?	
**Describe where the data may be found in full sentences. If you are copying our sample text, replace any instances of XXX with the appropriate details.**<br><br>• If the data are **held or will be held in a public repository**, include URLs, accession numbers or DOIs. If this information will only be available after acceptance, indicate this by ticking the box below. For example: *All XXX files are available from the XXX database (accession number(s) XXX, XXX.).*<br>• If the data are all contained **within the manuscript and/or Supporting Information files**, enter the following: *All relevant data are within the manuscript and its Supporting Information files.*<br>• If neither of these applies but you are able to provide **details of access elsewhere**, with or without limitations, please do so. For example:<br><br>*Data cannot be shared publicly because of [XXX]. Data are available from the XXX Institutional Data Access / Ethics Committee (contact via XXX) for researchers who meet the criteria for access to confidential data.*<br><br>*The data underlying the results presented in the study are available from (include the name of the third party*	All relevant data are within the manuscript and its Supporting Information files

and contact information or URL).<br>• This text is appropriate if the data are owned by a third party and authors do not have permission to share the data.<br><br>* typeset	
Additional data availability information:	

Manuscript Click here to access/download;Manuscript;Nadieline et al-April 2024.docx

Development of on-farm AMF inoculum production for sustainable agriculture in Senegal

Short title: On-farm AMF inoculum

Christian Valentin Nadieline[1], Antoine le Quéré[2], Cheikh Ndiaye[3], Amadou Abib Diène[4], Francis Do Rego[5], Oumar Sadio[6], Yoro Idrissa Thioye[7], Marc Neyra[2], Cheikh Mouhamed Fadel Kébé[8], Tatiana Krasova Wade[5]*

[1]Institut de Recherche pour le Développement (IRD), Laboratoire Commun de Microbiologie IRD/ISRA/UCAD (LCM), Centre de Recherche de Bel-Air, Dakar, Senegal ; christianvalentin.nadieline@ird.fr.

[2]Institut de Recherche pour le Développement (IRD), UMR Eco&Sols IRD/CIRAD/INRAE/Montpellier SupAgro, Campus de Baillarguet, Montpellier, France

[3]Département de Biologie Végétale, Faculté des Sciences et Techniques, Université Cheikh Anta Diop de Dakar, Dakar-Fann, Senegal.

[4]GIE Ndiénné, Commune de Darou Mousty, village Darou Mousty, Senegal.

[5]Institut de Recherche pour le Développement (IRD), UMR Eco&Sols IRD/CIRAD/INRAE/Montpellier SupAgro / IESOL, Centre de Recherche de Bel-Air, Dakar, Senegal.

[6]Institut de Recherche pour le Développement (IRD), UMR 195 LEMAR, ISRA-CRODT /Pôle de recherche de Hann, Dakar, Senegal.

[7]Conseil National de Concertation et de Coopération des Ruraux (CNCR), Dakar, Senegal.

[8]Ecole Supérieure Polytechnique de Dakar Département Génie Civil, Université Cheikh Anta Diop de Dakar, Dakar-Fann, Senegal.

*Corresponding author: Tatiana Krasova Wade, tania.wade@ird.fr

ORCID: https://orcid.org/0000-0002-2469-6415

Author contribution: Conceived and designed the experiments: TKW, CVN, ALQ. Performed the experiments: TKW, CN, CVN. Analyzed the data: CVN, CN, OS. Contributed

reagents/materials/analysis tools: AAD, FDR. Reviewed: ALQ, MN. Funding acquired: MFK, YIT, TKW. Project administered: TKW. Wrote the paper: TKW, CVN, ALQ.

Abstract

The integration of endomycorrhizal fungi into agricultural practices as inoculum offers the potential to enhance plant productivity while reducing the reliance on costly chemical fertilizers, which are not only economically onerous but also environmentally detrimental. Mycorrhizal fungi play a crucial role in facilitating plant access to essential mineral elements (such as Phosphorus, Potassium, etc.) and water, particularly in soils characterized by arid and semi-arid conditions where these resources are often limited. Despite the evident benefits, the production of arbuscular mycorrhizal fungi (AMF) inoculum in Africa remains constrained by technological limitations, with current operations typically confined to small or medium-scale endeavors. This research aims to address this limitation by exploring the feasibility of producing mycorrhizal inoculum on a semi-industrial farm scale, achieved through the control and stabilization of production parameters. Laboratory experiments were conducted to evaluate the multiplication of mycorrhizal fungi, including *Glomus aggregatum* IR27, *Funneliformis mosseae, Rhizophagus irregularis,* and *Glomus fasciculatum* R10. Various agricultural residues, such as peanut shells, rice husks, sugar cane bagasse and millet cobs, were tested as alternatives to conventional sand production supports. Significant results indicated that peanut shells can serve as a suitable substrate for upscaling and decentralizing production. Moreover, the study emphasized the critical considerations of irrigation water salt content and substrate sterilization as pivotal parameters to ensure optimal development of mycorrhizal propagules. This experimental endeavor serves as a pioneering initiative, laying the groundwork for the establishment of a local sector dedicated to the production and distribution of endomycorrhizal fungi inoculum in Senegal.

Key words: Arbuscular mycorrhizal fungi (AMF), Inoculum production, Agricultural residual products

Introduction

Agricultural activity within the West African sub-region is not sufficient to fulfill its raising population needs which also faces environmental changes. To address this challenges, commonly recommended solutions involve practices such as the application of chemical fertilizers, pesticide use, adoption of new seed varieties, and water control collectively referred to as agricultural intensification. However, these practices, while offering short-term gains, often result in long or medium-term degradation of resources and the environment, particularly contributing to groundwater pollution [1, 2].

Simultaneously, research outcomes have proposed various cultural practices aimed at restoring soil fertility levels and increasing plant production in developing countries in Africa [1]. Alternative strategies have been promoted to mitigate risks to agricultural production, which is already insufficient to meet the nutritional needs of populations, especially in developing countries. Among these strategies is the utilization of symbiotic microorganisms as inoculants, involving the multiplication of selected strains, particularly arbuscular mycorrhizal fungi (AMF) in the form of biostimulant inoculum [3].

Incorporating mycorrhizal fungi into agricultural practices represents a viable technical approach to enhance plant productivity while mitigating the need for costly and environmentally harmful chemical fertilizers. Through symbiosis, mycorrhizal fungi play a pivotal role in aiding plants to access mineral elements such as [illegible]osphorus and Potassium, as well as water resources frequently limited in arid and semi-arid soils [1, 4, 5, 6].

Mycorrhizal inoculation, as an ecological engineering technique developed through research, leverages the utilization of mycorrhizal fungi, natural symbiotic soil microorganisms. The mass application of a selected inoculum of proven quality has the potential to significantly enhance crop growth. This inoculum serves as a biological stimulant; it is cost-effective, environmentally friendly, and easy to use. Its application results in a reduction in the dependence on mineral fertilizers [7] and

contributes to the restoration of land fertility through the improved utilization of phosphorus and organic matter.

A meta-analysis encompassing 290 greenhouse and field trials aimed at investigating the impact of various agricultural practices on mycorrhizal colonization revealed that inoculation led to a substantial increase in mycorrhizal colonization, reaching up to 29% [2, 8]. Notably, in Senegal, research findings dating back to 1994 demonstrated the effectiveness of arbuscular mycorrhizal fungi (AMF) in root colonization, extending to depths of up to 40 meters in Sahelian zones [9]. Furthermore, mycorrhizal inoculation exhibited cost reduction and increased yields in cash crops [10], forestry [11, 12], and various food crops [13, 14, 15] across diverse agro-ecological zones. Additionally, it demonstrated adaptability to saline and water stress conditions [16].

While certain countries have witnessed significant progress in AMF production and utilization, Sub-Saharan African farmers face challenges due to limited production and awareness of the associated benefits, resulting in a lack of technological knowledge [1]. AMF inoculum production units are scarce in the region, with knowledge limited to Kenya and South Africa as of 2016 [1]. The production scale of mycorrhizal fungi inoculum in Africa remains modest due to technological constraints, underscoring the pivotal role of production technology and inoculum formulation support for sustainable application [17].

In Senegal, mycorrhizal inoculation has gained social acceptance among farmer organizations, agricultural advisory services, and management structures, creating a demand for its widespread availability [18, 19]. Recognizing this demand, the current challenge is the mass production of high-quality inoculum to meet growing needs, as research laboratories have historically produced inoculum on a small scale for research purposes.

This study endeavors to explore the feasibility of producing AMF inoculum on a semi-industrial scale within agricultural production sites. Knowledge transfer has been facilitated to farmer cooperatives specializing in seed production, offering them a new avenue to enhance production and diversify their

offerings. Transitioning from small-scale laboratory production to in situ inoculum production requires adapting the production process using cost-effective equipment. This approach should allow for technology replication in agricultural areas, minimizing distribution costs and transportation constraints. Ultimately, it contributes to the integration of symbiotic microorganisms into agricultural practices in the form of bio-stimulants.

The process of AMF inoculum production typically involves monoculture starters or strain mixtures under controlled conditions in greenhouses, plant growth chambers, or occasionally in open-air farm settings [19, 20, 21, 22, 23, 24]. Starters typically contain propagules in the form of spores, colonized root fragments, and hyphae [25, 26), and are multiplied on growing substrates such as peat, compost, vermiculite, sand, etc. [27]. These fungi, being obligate symbionts, necessitate host plant presence for cultivation. The most prevalent method for solid medium multiplication involves depositing colonized root fragments or AMF spores in a substrate sterilized at high temperatures and atmospheric pressure, or via gamma radiation. Subsequently, seeds of a plant capable of forming mycorrhizae are sown. After several months, the substrate and roots of the host plant can be used as inoculum [28]. The quality and effectiveness of the biostimulant during production and post-introduction into the soil are influenced by temperature, humidity, substrate pH, and the host plant, thereby potentially impacting crop yields [29, 30, 31, 32].

This study proposes a method for large-scale arbuscular mycorrhizal fungi (AMF) inoculum production directly in the field, utilizing locally available means and materials, with a focus on controlling and stabilizing production parameters. The objective is to establish a practical and sustainable approach that aligns with the local context, enabling widespread adoption and integration into existing agricultural practices.

The envisioned large-scale production method involves harnessing the natural environment while adhering to specific parameters critical for successful AMF inoculum development. By utilizing indigenous resources and optimizing the available infrastructure, this approach seeks to overcome

technological limitations and promote a cost-effective, environmentally friendly, and easily replicable process.

The key components of this field-based production method include the control of relevant factors essential for AMF proliferation such as substrate composition & treatment, watering and cultivation processes. This requires adaptation to local climatic conditions and the selection of appropriate growing substrates, ensuring compatibility with the target AMF strains. The method emphasizes the use of low-cost, locally sourced materials that can create a conducive environment for AMF multiplication.

Furthermore, the proposed approach involves community engagement and knowledge-sharing to empower local farmers and cooperatives. Training programs will be developed to disseminate the necessary expertise, fostering a sense of ownership and encouraging self-sufficiency in AMF inoculum production. By integrating this method into existing agricultural systems, it aims to promote sustainable farming practices and contribute to the overall enhancement of soil health and crop yields.

The success of this large-scale field-based AMF inoculum production method relies on its adaptability to diverse agro-ecological zones, emphasizing the importance of local context in the decision-making process. Through collaborative efforts between researchers, farmers, and agricultural extension services, the goal is to establish a model that not only meets the demand for high-quality inoculum but also contributes to the broader sustainability goals of agricultural development in the region.

Material and methods

Experimental sites

The experiments were conducted at the Joint Microbiology Laboratory IRD-ISRA-UCAD (LCM) located at the Bel Air Research Center in Dakar, Senegal, and in a greenhouse on a pilot site for mycorrhizal fungi inoculum production in the commune of Darou Mousty, Senegal.

Arbuscular mycorrhizal fungi (AMF) material

The initial mycorrhizal strains used for multiplication in the laboratory and on the pilot site (starters) were provided by the LCM. These strains included *Glomus aggregatum* IR27 [33], *Funneliformis mosseae* [30], *Rhizophagus irregularis* [34], and *Glomus fasciculatum* R10 [30]. The material consisted of a mixture of sand (as a culture substrate), fragments of mycorrhizal roots, spores, and AMF hyphae. The mycorrhization parameters of the initial strains, multiplied prior to multiplication on the pilot site, are presented in Table 1.

Table 1. Mycorrhization parameters (mean data) of AMF starters used for inoculum production on the pilot site

AMF Strain	Mycorrhization frequency (%)	Mycorrhization intensity (%)	Number of spores (100^{-1}g of substrate)
Glomus agregatum IR27	100	92	1313
Funneliformis mosseae	95	90	1024
Glomus fasciculatum	90	85	917
Rhizophagus irregularis	100	95	1230

AMF multiplication using agricultural residues as inoculum support in the lab

To explore the feasibility of substituting sand with agricultural residues from crop production, experiments were conducted in Leonard jars and pots in the laboratory. Various agricultural residues, such as peanut hulls, rice husks, millet ears, and sugarcane bagasse, were utilized either independently or in combination with sand (50/50, v/v). Prior to experimentation, these residues were crushed,

humidified, and sterilized in an oven at 65°C for three days. The sand was autoclaved at 120°C for 2 hours. Corn (*Zea mays* L.) was chosen as the host plant.

For multiplication, five grams (5 g) of a mixture containing the four AMF strains in equal proportions were used. The plants were irrigated with demineralized water. After three months of cultivation, watering was suspended for three weeks to induce stress in the plants and facilitate sporulation. The aerial parts of the corn plants were initially harvested, and the inoculum was subsequently collected from each container by blending the cut corn roots with the production support.

Inoculum quality control

Quality control of amplified AMF involved assessing two parameters: mycorrhization rate through corn root coloring and spore count by extraction and enumeration in the culture substrate.

In each sample, a portion of the corn roots was selected for coloring following the method of Philips and Hayman [35] to determine the mycorrhization rate. Briefly, the sampled roots were thoroughly rinsed in tap water to remove soil particles and then immersed in a 10% (w/v) KOH solution. KOH aided in discoloring the roots and emptying the cytoplasmic contents of the root cells. The tubes containing the roots and KOH were boiled in a water bath at 90°C for one hour. Subsequently, the roots were thoroughly rinsed to eliminate KOH and immersed in a 0.05% (w/v) Trypan blue solution for staining in a water bath at 80°C for 30 minutes. Following this step, the roots were washed with water and stored in a cool place for histological observation. For each sample, approximately 1 cm-long root fragments were interposed between a slide and coverslip, subsequently crushed in glycerol, and subjected to microscopic observation. The identification of hyphae, vesicles, or arbuscules in the root enables the estimation of the colonization rate in the root sample. Arbuscular mycorrhizal fungi root colonization was assessed using the method outlined by Trouvelot et al. [36].

Spore extraction and quantification were conducted following the protocol established by Gerdemann and Nicolson [37]. Initially, 100 g of inoculum was combined with 1 liter of tap water, followed by

stirring for 1 minute and settling for 30 seconds. The supernatant underwent filtration through a series of stacked sieves with diminishing mesh diameters (400 µm, 200 µm, 100 µm, 50 µm). Spores retained by the 200 µm and 50 µm sieves were gathered in distilled water and transferred to centrifugation tubes. Subsequently, two sucrose solutions (20% and 60%, v/v) were sequentially introduced into the tubes using a pipette. Centrifugation was carried out at 3000 RPM min^{-1} for 3 minutes at 4°C. The spores in each sample were recovered at the interface of the two sucrose solutions using a pipette and thoroughly rinsed with distilled water through a 50µm sieve. Finally, spores obtained after extraction were enumerated under a binocular magnifying glass.

Scaling up mycorrhizal inoculum production on peanut shells at the pilot site

Crushed peanut shells were used as substrate at the pilot site. The shells were moistened with tap water and sterilized by cooking for 30 minutes in metal barrels heated with firewood, by reference with the sand whose sterilization in the same system for 60 min made it possible to obtain the complete destruction of the spores (d[illegible] not shown). The sterilized material was then distributed into plastic containers (54 cm x 80 cm); each container received 12.5 kg of sterile crushed peanut shells. Five treatments were tested. These included the four AMF starters provided by LCM and one control non-inoculated treatment. Corn (*Zea mays* L.) served as the host plant, with seeds soaked in tap water for 2 hours before being directly sown at a rate of 20 seeds per container. In each container, a total of 200 g of microbial starter was deposited at a depth of 2-3 cm. Each treatment comprised twelve repetitions.

The multiplied inoculum was collected at the end of the growth phase after 3 months of cultivation and 3 weeks off watering. Aerial parts of the corn were removed, and the inoculum was harvested from each container by mixing thoroughly cut corn roots with the peanut shell substrate. Subsequently, a 100 g sample from each repetition and each treatment was collected and combined

with eleven other aliquots from the same treatment to create a composite inoculum for quality control at the laboratory in Dakar. Analysis of the chemical composition of the peanut shell, both before and after inoculum production, was conducted at the Laboratory of Analytical Means in Dakar (http://www.imago.ird.fr/).

Quality control of the inoculum produced on peanut shells at the pilot site

Quality control consisted in estimating the mycorrhization rate as previously described. The spore extraction protocol from the peanut shell was however adapted, incorporating a preliminary step to remove plant debris. This phase involved placing 100 g of inoculum in 2 L of tap water in a container, stirring the mixture, and pouring it through a 500 µm sieve. This debris removal process was repeated three times. Once largest debris discarded, the protocol continued with the Gerdemann and Nicolson [37] method, spooning the water content (6 L) through a series of sieves (400, 200, 100, and 50 µm). In each case, spores retained by the 200 µm and 50 µm sieves were collected in distilled water and transferred to centrifuge tubes. The subsequent steps of the protocol were then followed as described before. The spores obtained after extraction were counted under a binocular magnifying glass.

Effect of salt in watering water and substrate sterilization on inoculum quality

To find out whether we could use water from the Darou Mousty site, which contains salt, and a non-sterile substrate, we assessed the impact of the salt content of the irrigation water and of substrate sterilization on the quality of the inoculum produced in a new experimental design under laboratory conditions. Starter inoculum from LCM, comprising a mixture of the four strains in equal proportion,

was used for this test. The inoculum was applied to sown corn on peanut shells under the following treatments in triplicates:

• Unsterilized substrate, inoculated (with the 4 AMF strains) and watered with unsalted water (LCM*NoSalty*No Sterile);

• Unsterilized substrate, inoculated and watered with salt water (LCM*Salty*No Sterile);

• Sterilized substrate, inoculated and watered with unsalted water (LCM*No Salty*Sterile);

• Sterilized substrate, inoculated and watered with salt water (LCM*Salty*Sterile);

• Sterilized support, not inoculated and watered with salt water (T*Salty*Sterile);

• Unsterilized support, uninoculated and watered with unsalted water (T*No Salty*No Sterile);

• Sterilized support, not inoculated and watered with non-salty water (T*No Salty*Sterile);

• Unsterilized support, uninoculated and watered with salt water (T*Salty*NoSterile).

In each case, the substrate consisting in sterilized or non-sterilized shells was distributed among 6 jars of 2 L each, and received ten grams of the AMF starter mixture as inoculum. Three pots were watered with drinking water, and three with a salted solution of 0.5% (w/v) of dietary salt (corresponding to salt concentration found in Darou Mousty irrigation water). Five corn seeds were sown into each pot.

Statistical analyses

For the CMA production test on different agricultural residues in Leonard pots and jars, the effects of inoculation and support on the intensity of mycorrhization, the frequency of mycorrhization, and the number of spores were analyzed. Firstly, the normality of the data distribution for all variables was assessed using the Shapiro-Wilk test for each combination of factor levels. Subsequently, homogeneity of variance was evaluated. The effect of inoculation was assessed using a two-way analysis of variance (ANOVA) for the jar test and a three-way analysis of variance (ANOVA) for the Leonard jar test. To evaluate the salinity effect of salted water on inoculum quality, the same analysis

as before (normality, homogeneity of variance) was conducted, and a three-way analysis of variance was employed to assess the effect. Statistical significance was considered at a level of $p < 0.05$. A Principal Component Analysis (PCA) was also constructed for this design. All statistical analyses were performed using R software (R Core Team, 2023) [38].

Results

Effect of agricultural residue supports on the production of AMF inoculum

The data analysis revealed a significant impact of corn inoculation on mycorrhization parameters, including intensity, frequency, and spore count, in agricultural residues, both within Leonard jars (Table 2) and pots (Table 3), as compared to the control.

Table 2. ANOVA of the mycorrhization parameters obtained on agricultural residues in Leonard jars

**Mycorrhization intensity**	Sum Sq	Mean Sq	F value	Pr(>F)	
Substrat	1007	252	2.191	0.127	
Inoculation	3672	3672	31.944	7.9e-05	***
Substrat	140	140	1.222	0.289	
Residuals	1494	115			
**Mycorrhization frequencies**					
Substrat	2111	528	2.285	0.116	
Inoculation	14098	14098	61.048	2.89e-06	***
Substrat	76	76	0.329	0.576	
Residuals	3002	231			
**Number of spores**					

Substrat	341123	85281	3.108	0.0533	.
Inoculation	846250	846250	30.845	9.32e-05	***
Substrat	5645	5645	0.206	0.6576	
Residuals	356665	27436			

Table 3. ANOVA of the mycorrhization parameters obtained on agricultural residues in pots

**Mycorrhization intensity**	Sum Sq	Mean Sq	F value	Pr(>F)	
Substrat	556.8	139.2	1.00	0.5000	
Inoculation	2312.8	2312.8	16.62	0.0151	*
Residuals	556.8	139.2			
**Mycorrhization frequencies**					
Substrat	1905	476	1.00	0.500	
Inoculation	8294	8294	17.42	0.014	*
Residuals	1905	476			
**Number of spores**					
Substrat	99959	24990	1.00	0.5000	
Inoculation	354569	354569	14.19	0.0197	*
Residuals	99959	24990			

No statistically significant differences were observed among residues and upon addition of sand both under controlled conditions (Table 2) and under semi-controlled conditions (Table 3). However, the most favorable characteristics of the inoculum were consistently achieved on sand, peanut shell alone, or a combination of peanut shell and sand both under controlled or semi-controlled conditions (respectively Tables 4 and 5).

Table 4. Characteristics of the inoculum produced on agricultural residues alone or mixed with sand in Leonard jars

Substrate*	Mean Mycorrhization intensity (%)	Mean Mycorrhization frequencies (%)	Mean Number of spores (100^{-1}g of Substrate)
peanut shell	53,9	90	712
rice husk	29,7	62	543
ears of millet	16,9	44	271
bagasse	7,1	25	18
sand	54,4	64	681
Substrate/sand (50/50)			
peanut shell	29,2	55	639
rice husk	30,5	78	537
ears of millet	15,8	51	101
bagasse	4,3	7	21

*Uninoculated control plants were grown on each substrate and showed no mycorrhizal development.

Table 5. Characteristics of the inoculum produced on agricultural residues in pots

Substrate*	Mean Mycorrhization intensity (%)	Mean Mycorrhization frequencies (%)	Mean Number of spores (100^{-1}g of Substrate)
peanut shell	46,3	86	527
rice husk	37,4	84	503
ears of millet	20,48	31	278
bagasse	6,2	19	27
sand	41,7	68	548

*Uninoculated control plants were grown on each substrate and showed no mycorrhizal development.

Peanut shell and sand exhibited superior performances for most mycorrhization parameters assessed: mycorrhization intensity, mycorrhization rate, and spore count, both in Leonard Jars and pots. Consequently, peanut shell was chosen as sustainable substrate to test the scaling up of inoculum production into a delocalized pilot site in Darou Mousty.

Scaling-up production of mycorrhizal fungi inoculum on peanut shell at the farm in the rural zone

The mycorrhization parameters of the mass-produced inoculum on the peanut shell are presented in Table6.

Table 6. Characteristics of the inoculum produced on the peanut shell at the pilot site

		G. agregatum IR27	*G. fasciculatum*	*F. mosseae*	*R. irregularis*	Control
Mean Number of spores (100^{-1}g substrate)	Average	**13,4 a**	**15,4 a**	**13,6 a**	**21,2 a**	**6,2 a**
	Std	5,5	13,48	1,34	15,06	7,12
	Repeat	5	5	5	5	5
	Min	5	4	12	5	0
	Max	20	34	15	38	17
Mean Mycorrhization intensity (%)	Average	**19,76 a**	**13,66 ab**	**8,66 bc**	**9,58 b**	**0,91 c**
	Std	6,55	5,95	2,84	4,12	0,81
	Repeat	5	5	5	5	5
	Min	8,3	6	5,3	5,3	0,2
	Max	24	22,5	12,2	15	2,1
Mean Mycorrhization frequency (%)	Average	**88,93 a**	**70,4 a**	**62,07 ab**	**42,20 bc**	**15,0 c**
	Std	5,36	16,79	18,23	17,21	20,55
	Repeat	5	5	5	5	5
	Min	83,33	43,33	45	20	0
	Max	96,67	86,67	85	68,33	38,33

The statistical analysis revealed that the number of spores produced by mycorrhizal fungi did not exhibit significant differences between the strains, nor when compared to the control. However, the mycorrhization intensity demonstrated a highly significant difference (Pr=8.07e-05), with the strains *Glomus aggregatum*, *Glomus fasciculatum*, and *Rhizophagus irregularis* (in descending order) displaying superior performance compared to the control. Notably, no significant distinction was observed between the *Glomus aggregatum* and *Glomus fasciculatum* strains regarding this parameter.

Regarding mycorrhization frequencies, the strains *Glomus aggregatum*, *Glomus fasciculatum*, and *Funneliformis mosseae* exhibited significantly enhanced performance (in descending order) compared to the control (Pr=3.28e-05).

However, when compared with the characteristics of the starters used for inoculum production (Table 1), such as the number of spores (1313 versus 13.4 for the *Glomus aggregatum* IR27 strain, for instance) and the intensity of mycorrhization (95% versus 8.66% for *Rhizophagus irregularis*), the infectious potential of the inoculum exhibited very low values (Table 6). Among the parameters potentially influencing inoculum quality, factors such as the chemical composition of peanut shell (particularly phosphorus content), quality of irrigation water, or the spore extraction method were investigated. Chemical analyses of the peanut shell composition before and after production did not reveal any significant differences (Table 7). Minor increases in carbon and phosphorus contents were noted, along with slight decreases in sodium and potassium contents, pH, and conductivity in the inoculum.

Table 7. Chemical composition of peanut shell before and after the inoculum production (mean values)

Peanut shell	total N (%)	total C (%)	total P (mg kg^{-1})	Na (mg kg^{-1})	K (mg kg^{-1})	pH	Conductivity (µS/cm)
Before production	0.79	38.98	575	2 566.0	1 632	6.04	0.42
After production	0.79	43.21	602.67	2 322.0	1 545.67	5,81	0.38

On the other hand, concerning the water utilized for irrigating the corn crop during inoculum production, the salt concentration was measured using a salinometer and registered a value of 5 g/L. According to a classification of salinity limits for irrigation water [39], this concentration corresponds

to 0.78 dS/m and is considered as having an average salinity level. To evaluate the potential impact of water salinity on inoculum quality, another experiment was conducted in the laboratory as described in Materials and Methods section.

Effect of irrigation water salinity and sterilization on the quality of mycorrhizal inoculum produced using peanut shells

The results of the 3-way ANOVA showed a significant interaction Substrate*Water*Treatments on the variables intensity and frequency of mycorrhization, and the interactions Substrate*Treatments and Water*Treatments on the variable number of spores (Table 8).

Table 8. **Type III sums of squares analysis (Spores, Intensity and frequency)**

Parameters		ddl	Sum of squares of deviations	Mean square	F Value	Pr(>F)
Spores	Substrate	1	10127	10127	13.032	0.002842**
	Water	1	11137	11137	14.332	0.002007**
	Treatments	1	53865	53865	69.316	8.58e-07***
	Repetitions	2	2283	1141	1.469	0.263625
	Substrate*Eau	1	672	672	0,865	0.368156
	Substrate*Treatments	1	21780	21780	28.028	0.000113 ***
	Water*Treatments	1	6767	6767	8.708	0.010526 *
	Substrate*Water*Treatments	1	2501	2501	3.218	0.094432
	Residuals	14	14 10879	777		
Intensity	Substrate	1	14,122	14,122	19,137	0.000635 ***
	Water	1	25,072	25,072	33.976	4.38e-05 ***
	Treatments	1	15,958	15,958	21.625	0.000375 ***
	Repetitions	2	0,324	0,324	0.219	0.805865
	Substrate*Water	1	15,763	15,763	21,361	0.000396 ***

	Substrate*Treatments	1	24,827	24,827	21.361	0.000396 ***
	Water*Treatments	1	14,307	14,307	19.388	0.000601 ***
	Substrate*Water*Treatments	1	26,988	26,988	19.388	0.000601 ***
	Residuals	14	10,331	0,738		
Frequency	Substrate	1	234.1	234.1	129.759	1.82e-08 ***
	Water	1	550.9	550.9	305.431	6.64e-11 ***
	Treatments	1	550.9	550.9	305.431	6.64e-11 ***
	Repetitions	2	0.3	0.1	0.082	0.922
	Substrate*Water	1	301.0	301.0	166.851	3.61e-09 ***
	Substrate*Treatments	1	301.0	301.0	166.851	3.61e-09 ***
	Water*Treatments	1	458.9	458.9	254.424	2.25e-10 ***
	Substrate*Water*Treatments	1	376.3	376.3	208.600	8.39e-10 ***
	Residuals	14	25.3	1.8		

The inoculum obtained on peanut shells from the LCM starter, watered with unsalted water, exhibited a superior mycorrhization profile (frequency: 33.33% and intensity rate: 7.33%). Notably, all samples watered with salty water displayed an absence of mycorrhization, except for the inoculum obtained from the LCM starter on the non-sterile substrate, attributed to the presence of exogenous strains. The sterile support yielded better results than the non-sterile one for frequency, intensity, and spore count (mean of 211 spores/100g of inoculum collected on sterile support compared to a mean of 79 spores/100g of inoculum collected on non-sterile support). The detrimental impact of saline irrigation water on propagule development was confirmed through mycorrhization parameters of control plants grown on non-sterile support in the presence of exogenous AMF fungi (Fig 1).

Fig 1. Box plot showing the effect of using salt water and substrate sterilization on inoculum quality parameters (Spore frequency, mycorrhization Intensity and Frequency). *LCM refers to inoculation with the mix of LCM strains; T, non-inoculated; Salty, irrigated with salted water (NaCl, 0.5% W/V); No_Salty, irrigated with unsalted water; Sterile, sterilized peanut shell support; Non_Sterile, unsterilized peanut shell support.*

Principal component analysis (PCA) carried out on the mycorrhization parameters (number of spores, mycorrhization frequency and intensity) in relation to the substrate (sterile and non-sterile) and to the irrigating water (salty and non-salty) showed that 99.5% of variance were explained by the first 2 principal components (PC1:93.7% and PC2:5.8%) (Fig 2).

Fig 2. Principal Component Analysis showing the effects of the treatments on the mycorhization using as variables the number of spores, mycorrhization intensity and frequency. *LCM*No Salty*No Sterile: unsterilized support, inoculated and watered with unsalted water; LCM*Salty*No Sterile: unsterilized support, inoculated and watered with salt water; LCM*No Salty*Sterile: sterilized support, inoculated and watered with unsalted water; LCM*Salty*Sterile: sterilized support, inoculated and watered with salt water; T*Salty*Sterile: sterilized support, not inoculated and watered with salt water; T*No Salty*No Sterile: unsterilized support, uninoculated and watered with unsalted water; T*No Salty*Sterile: sterilized support, not inoculated and watered with non-salty water and T*Salty*No Sterile: unsterilized support, uninoculated and watered with salt water.*

The analysis clearly separates on the first axis the treatment LCM*No Salty*Sterile from other treatments (LCM*Salty*Sterile, LCM*No Salty*No Sterile, LCM*Salty*NoSterile, T*Salty*Sterile, T*No Salty*No Sterile, T*No Salty*Sterile and T*Salty*No Sterile) that appeared homogeneous on the first axis and which were also associated to lower mycorrhization capacity. The PCA results clearly showed that the LCM*No Salty*Sterile treatment stood out with a better mycorrhizal potential (spores, intensity and frequency) (Fig 2). These results demonstrate the importance to sterilize the substrate but also the negative effect of salinity on the mycorrhization potential using peanut shells as substrate.

The results of the experiments are shown as box plots (Fig 1) and clearly show that watering the corn with unsalted water improve all mycorrhization parameters. Water containing 0.5% salt inhibited mycorrhization. This negative effect of salt was much more accentuated in unsterilized peanut shell substrate than in sterilized one (Fig 1).

Improved method for extracting mycorrhizal spores from peanut shell substrate

Sterilization of the peanut shell by cooking for 30 minutes with firewood in the field led to acquiring a support with a significantly lower spore count compared to control plants (refer to Table 6). Furthermore, an enhancement in the spore extraction method applied to the inoculum produced on peanut shells was achieved compared to the classic method on sand. The Fig 3 presents the results obtained using the two methods. The improved method resulted in collecting a significantly higher number of spores ($F=21.88$; $p<0.0001$) both in the inoculum obtained in the lab from LCM starter (mean of 123.6 spores collected by the improved method vs. 106 by the classic method) and in the inoculum produced in Darou Mousty (81 spores collected by the improved method vs. 45 spores by the classic method).

Fig 3. Mean number of spores obtained by classical and improved extraction methods when using peanut shells as growth substrate. *The means of three repetitions per treatment followed by different letters differ according to the Fisher test (p = 0.05). Bars represent standard deviations from the mean (n=3). The comparison is made within each treatment. INO, inoculum originated from the Darou Mousty pilot site obtained with salted and unsalted irrigation water; LCM, inoculum obtained from starter of Joint Microbiology Laboratory (LCM) on peanut shells in the laboratory; control, uninoculated corn grown in peanut shell*.

Discussion

This study demonstrates the potential for scaling up the multiplication of arbuscular mycorrhizal fungi (AMF) inoculum beyond the confines of a semi-controlled laboratory environment. Agricultural residues, as opposed to the commonly used sand, emerge as a viable alternative for supporting inoculum production. Heavy, sand as a conventional support, poses challenges when transitioning to large-scale production, while numerous locally available and annually renewable organic materials

from agricultural production stand as advantageous alternatives. In this study, peanut shell emerged as a promising substrate, undergoing partial decomposition during inoculum production due to corn growth, it may also subsequently contribute to soil amendment and restitution of organic matter (Carbon, Phosphorus, and Nitrogen) upon field inoculation. Its use suggests potential accessibility of these elements to the inoculated cultures.

The refinement of the spore extraction protocol from peanut shell, involving the incorporation of a 500 µm sieve for enhanced spore passage, resulted in improved inoculum characteristics. Numerous and lightweight plant debris, obstructing spore passage through the initial sieve, were effectively addressed, leading to a significant increase in spore quantity collected in subsequent sieves.

Several authors [1, 40] have enumerated key characteristics for an ideal inoculum support, encompassing good water retention capacity, optimal dry matter content, carbon, pH, porosity, ease of sterilization and adjustment, non-toxicity, and availability. Studies by Saranya and Kumutha and Coelho et al. [41, 42] have emphasized the positive impact of adding organic matter to sand as a substrate for sporulation promotion, with optimal benefits dependent upon the specific organic source. The highest spore production was reported when vermicompost constituted 10% of the substrate [41] or when a mixture of sand, vermiculite, and 10% vermicompost was employed [42]. According to Saranya and Kumutha [41], compost has been identified as an excellent alternative substrate for mycorrhizal fungi multiplication, and biochar has also been recommended for large-scale production due to its influence on nutrient availability and organic matter stabilization. Further studies combining peanut shells enriched in compost or biochar that can be produced and acquired locally may further improve AMF inoculum quality in the future.

Nevertheless, the selection of agricultural residues suitable for AMF production necessitates consideration of phosphorus content, given its influential role in regulating AMF propagule production [43]. Douds Jr [44] observed increased sweet potato yields in soils with high phosphorus content when using an inoculum containing a mixture of *Glomus intraradices* strains. Similarly, the

addition of phosphorus to the irrigation solution of *P. miliaceum* plants promoted *G. etunicatum* sporulation, highlighting the potential adaptability of strains to high phosphorus conditions.

Among the studied strains, *Rhizophagus irregularis* exhibited significantly better sporulation under pilot site culture conditions, although not differing significantly from the control. In contrast, *Glomus agregatum* IR27 and *Glomus fasciculatum* showed significantly improved characteristics in terms of mycorrhization intensity (19.76% and 13.66%, respectively) and frequencies (88.93% and 70.4%, respectively), when compared to the control. The detrimental impact of salt in irrigation water on mycorrhizal propagule development and corn root colonization, previously established in controlled laboratory conditions, was confirmed. Among commercially available products, R. *irregularis* and F. *mosseae* are the most used species, with R. *irregularis* prevailing in 39% of 68 products from 28 companies [46].

This experience validated the selection of a support meeting the criteria of availability, adaptability to plant and fungal development, and the ability to ensure high-quality inoculum. The results underscore the significance of substrate sterilization and the quality of irrigation water as critical parameters for obtaining superior-quality inoculum. Salomon et al. [32] proposed a fundamental framework for quality control of commercial inocula, addressing essential criteria and control measures such as spore counts, root colonization, absence of pathogens eliminated through substrate sterilization, and support quality. A positive growth response in host plants and significant arbuscular mycorrhizal (AM) root colonization, with at least 20% of the root length colonized, are crucial indicators of inoculum viability.

Despite the central role of bio-inoculums in sustainable agriculture, the production and adoption of AMF in smallholder systems in Africa remain limited due to a lack of awareness and understanding. Research capacity and technological challenges, as identified by Mukhongo et al. [1], constitute significant barriers. Our study demonstrates the feasibility of transferring technology and know-how

for the on-farm production of AMF inoculum from the laboratory to the field. This adaptation utilizes locally available equipment and aligns with the scale and technical capacity of the local context.

On-farm production of AM fungus inoculum emerges as a viable option in labor-intensive agricultural settings typical of developing countries, utilizing materials readily accessible to farmers [47]. This approach offers cost savings by eliminating associated processing and shipping expenses included in the price of imported bioferilizers. The demonstrated yield increases, in conjunction with potential economic returns for farmers and environmental benefits resulting from reduced fertilizer and pesticide use, position on-farm production of AMF inoculum as an attractive alternative to importing inoculum [1]. The availability of molecular tools and markers for monitoring mycorrhizal fungi in roots and soil now supports microbiological analyses to verify their establishment [48, 32]. However, the effectiveness of isolates is not always directly linked to the degree of mycorrhizal colonization, necessitating field testing of inoculum effectiveness on local crops. Finally, this system can now easily be tested on alternative host plants and for the selection, amplification and conservation of native AMF strains which would participate in the preservation and the valorization of local biodiversity of this important group of plant beneficial microbes.

Conclusion

A technical package for scaling up the production of Arbuscular Mycorrhizal Fungi inoculum was developed in the field. The production protocol under the tested conditions and the quality control protocol for the produced inoculum were optimized. Management of inoculum production by farmers and the local availability of biological fertilizers are essential for sustainable agriculture while respecting the environment. Based on this experience, the establishment of a local sector for the production and distribution of endomycorrhizal fungi inoculum in Senegal appears feasible. Validated in a delocalized site, this optimized process not only addresses the technical challenges and constraints of traditional methods but also emphasizes the adaptability of the process to local

conditions. This innovative approach facilitates the availability of high-quality AMF inoculum for smallholding farmers, offering a sustainable and cost-effective solution to enhance crop productivity, reduce reliance on external inputs, and promote environmentally friendly agricultural practices. Finally, its use may participate in the preservation and valorization of AMF biodiversity by farmers cooperatives which could participate in the selection of locally adapted strains which the research partners could now easily characterize using sequencing technologies and control.

Acknowledgments:

This work was supported by the African Intellectual Property Organization (OAPI) funding 27714 and the Senegalese Innovative Business Incubator (INNODEV) between 2013 and 2016, and by the French National Research Institute for Sustainable Development (IRD) funding MySen between 2017 and 2019. The authors thank the management of the Joint Microbiology Laboratory IRD-ISRA-UCAD (LCM) for supporting and the authorities of the commune of Darou Mousty and the farmers for the facilities granted to this work.

References

1. Mukhongo R. W., Tumuhairwe J. B., Ebanyat P., Abdel Gadir A. H., Thuitaand M., Masso C. (2016). Production and use of arbuscular mycorrhizal fungi inoculum in sub-Saharan Africa: Challenges and ways of improving. *Int. J. Soil Sci.*, 11, 108-122. DOI: 10.3923/ijss.2016.108.122.
2. Oruru M. B. & Njeru E. M. (2016). Upscaling Arbuscular Mycorrhizal Symbiosis and Related Agroecosystems Services in Smallholder Farming Systems. *BioMed Research International*, ID 4376240. http://dx.doi.org/10.1155/2016/4376240.
3. Martín F. F., Molina J. J., Nicolás E. N., Alarcón J. J., Kirchmair M., F. J. García, et al. (2017). Application of Arbuscular Mycorrhizae *Glomus iranicum* var. *tenuihypharum* var. *nova* in

Intensive Agriculture: A Study Case. *J. Agricultural Science and Technology* B 7, 221-247. DOI: 10.17265/2161-6264/2017.04.001.

4. Auge R. M. (2001). Water relations, drought and vesicular-arbuscular mycorrhizal symbiosis. *Mycorrhiza*, 11, 3-42. DOI : 10.1007/s005720100097.
5. Duponnois R., Colombet A., Hien V., Thioulouse J. (2005). The Mycorrhizal fungus *Glomus intraradices* and rock phosphate amendment influence plant growth and microbial activity in the rhizosphere of *Acacia holosericea*. *Soil Biol. Biochem.*, 37, 1460-1468. DOI: 10.1016/j.soilbio.2004.09.016.
6. Lambers H., Raven J.A., Shaver G. R., Smith S.E. (2008). Plant nutrient-acquisition strategies change with soil age. Trends in Ecology. *Evolution*, 23: 95-103. DOI: 10.1016/j.tree.2007.10.008.
7. Sow H., Diop T. 2004. Champignons mycorhiziens: un potentiel pour un maraîchage rentable et adapté à la bourse des producteurs. *Info Agri Afrique*. 3-4, 27-29.
8. Lekberg Y. & Koide R. T. (2005). Is plant performance limited by abundance of arbuscular mycorrhizal fungi? A meta-analysis of studies published between 1988 and 2003. *New Phytologist*, 168, 189-204. DOI: 10.1111/j.1469-8137.2005.01490.x.
9. Diop T. A., Gueye M., Dreyfus B. L., Planchette C., Strullu D. G. (1994). Indigenous Arbuscular Mycorrhizal Fungi Associated with *Acacia albida* Del. in different areas of Senegal. *AEM*, 60, 9, 3433-3436.
10. Sow H., Diop T. A., Ndiaye F., Manga A. G. B., Diallo A. (2008). Influence de la mycorhization arbusculaire sur la culture intensive de l'oignon (*Allium cepa*) au Sénégal. *Journal des Sciences*, 8, (1), 1-6.
11. Duponnois R., Plenchette C., Prin Y., Ducousso M., Kisa M., Bâ A.M. et al. (2007). Use of mycorrhizal inoculation to improve reafforestation process with Australian Acacia in Sahelian ecozones. *Ecological engineering*, 29, 105-112.

12. Thioye B., Hervé Sanguin H., Kane A., Sergio Mania de Faria S. M., Fall D., Prin Y. et al. (2019). Impact of mycorrhiza-based inoculation strategies on *Ziziphus mauritiana* Lam. and its native mycorrhizal communities on the route of the Great Green Wall (Senegal). *Ecological Engineering*, 128, 66–76.
13. Diop T. A., Fall-Ndiaye M. A., Diagne-Leye G., Sy-Ndir M., Kane A., Diallo A. (2005). Atouts et avantages des champignons pour une agriculture durable. *Agridape*, 28-30.
14. Diop I., Kane A., Krasova Wade T., Sanon K. B., Hougnandan P., Neyra M., et al. (2013). Impacts des conditions pédoclimatiques et du mode cultural sur la réponse du niébé (*Vigna unguiculata* L. Walp.) à l'inoculation endomycorhizienne avec *Rhizophagus irregularis*. *J. Appl. Biosci*. 69:5465 – 5474.
15. Do Rego F. A, Diop I., Sadio O., Da Sylva M. C., Agbangba C. O., Touré O. et al. (2015). Response of cowpea to symbiotic microorganisms inoculation (arbuscular mycorrhizal fungi and rhizobium) in cultivated soils in Senegal, *UJPS*, 3(2), 32-42.
16. Manga A., Ndiaye F., Sow H.A., Diallo A., Diop T. A. Des champignons mycorhiziens pour mieux supporter la salinité. *Senesylva*, 2006; 10.
17. Pal S., Singh H. B., Farooqui A., Rakshit A. Commercialization of arbuscular mycorrhizal technology in agriculture and forestry. In *Agriculturally Important Microorganisms: Commercialization and Regulatory Requirements in Asia*; Springer: Singapore, 2016; pp.97-105.
18. Sow H. A. & Neyra M. (2008). Paysans et chercheurs ensemble pour l'intégration des microorganismes dans le système agricole ouest africain, *AGRIDAPE*, 24, 20-22.
19. Le Quéré A., Krasova Wade T. (2019). Insights from Senegal: involving farmers in research is key to boosting agriculture. The Conversation (https://theconversation.com/insights-from-senegal-involving-farmers-in-research-is-key-to-boosting-agriculture-128085).

20. Dalpé Y. (2005). Les mycorhizes: un outil de protection des plantes mains non une panacée. *Phytoprotection*, 86, 53-59.
21. Douds D. D, Millner P. D. (1999). Biodiversity of arbuscular mycorhizal fungi in agroecosystems. *Agriculture, Ecosystems and Environment*, 74, 77-93.
22. Douds D. D. Jr., Nagahashi G., Pfeffer P. E., Kayser W. M., Reider C. (2005). On-farm production and utilization of arbuscular mycorrhizal fungus inoculum. *Can. J. Plant Sci.*, 85, 15-21. DOI:10.4141/P03-168.
23. Douds D. D. Jr., Nagahashi G., Pfeffer P. E., Reider C., Kayser W. M. (2006). On-farm production of AM fungus inoculum in mixtures of compost and vermiculite. *Biores Tech*. 97:809–818. DOI:10.1016/j.biortech.2005.04.015.
24. Gaur A., Adholeya A. (2002). Arbuscular-mycorrhizal inoculation of five tropical fodder crops and inoculum production in marginal soil amended with organic matter. Biol. Fertil. Soils 35, 214-218. DOI:10.1007/s00374-002-0457-5.
25. Chave M., Dufils A., Paut R., Perrin B., Tchamitchian M. Fiche technique (2) : Multiplier des champignons mycorhiziens sur son exploitation. 2017; 4. hal-02791120.
26. Dalpé Y.M &onreal M. (2004). Arbuscular mycorrhiza inoculum to support sustainable cropping systems. Crop Manag. 3, 1-11. DOI:10.1094/CM-2004-0301-09-RV.
27. Okon Y. & Hadar H. (1987). Microbial inoculants as crop-yield enhancers. *Crit. Rev. Biotechnol.* 6, 61–85.
28. IJdo M., Cranenbrouck S., Declerck S. (2010). Methods for large-scale production of AM fungi: past, present, and future. *Mycorrhiza*, DOI: 10.1007/s00572-010-0337-z.
29. Brundrett M., Bougler N., Dell B., Grove T., Malajczuk N. Working with Mycorrhizas in Forestry and Agriculture. Australian Centre for International Agricultural Research. Canberra, Australia, 1996; 344.

30. Diop, T. A., Simoneau, P., Dalpé, Y., Plenchette, C., Strullu, D. G. (1998). Biodiversité et variabilité génétique des *Glomales* associés à *Acacia albida* au Sénégal. *L'acacia au Sénégal*, 405-422.

31. Herrmann L. & Lesueur D. (2013). Challenges of formulation and quality of biofertilizers for successful inoculation. *Appl. Microbiol. Biotechnol.* 97, 8859-8873. DOI:10.1007/s00253-013-5228-8.

32. Salomon M. J., Watts-Williams S. J., McLaughlin M. J., Bücking H., Singh B. K., Hutter I. et al. (2022). Establishing a quality management framework for commercial inoculants containing arbuscular mycorrhizal fungi. i*Science* 25, 104636, July 15. https://doi.org/10.1016/j.isci. 2022.104636.

33. Bâ, A. M., Dalpé, Y., & Guissou, T. (1996). Les Glomales d'*Acacia holosericea* et d'*Acacia mangium*. *Bois & Forêts des tropiques,* 250, 5-18.

34. Dupponois R. & Plenchette C. (2003). A mycorrhiza helper bacterium enhances ectomycorrhizal and endomycorrhizal symbiosis of Australian *Acacia* species. *Mycorrhiza*, 13, 85-91.

35. Philips and Hayman. 1970. Improved procedures for cleaning roots and staining parasitic and vesicular arbuscular mycorrhizal fungi for rapid assessment of infection. Transactions British. *Mycological Society*, 55; 93-130.

36. Trouvelot A., Kough J. L., Gianinazzi-Pearson V. Mesure du taux de mycorhization VA d'un système radiculaire. Recherche de méthodes d'estimation ayant une signification fonctionnelle. *Mycorrhizae* : Physiology genetics. ESM Dijon, INRA Paris, 1985; 1-5.

37. Gerdemann J.W., Nicholson T.H. (1963). Spores of mycorrhizal endogone species extracted from soil by wet sieving and decanting.Trans. *Br. Mycol. Soc.* 46, 235-244.

38. R CORE TEAM, 2023. R : un langage et un environnement pour le calcul statistique. R Foundation for Statistical Computing, Vienne, Autriche. https://www.R-project.org/.

39. Lacharme M. Fascicule 9 du mémento technique de riziculture, le contrôle de la salinité dans les rizières. 2001; 20.

40. Herrmann L & Lesueur D. (2013). Challenges of formulation and quality of biofertilizers for successful inoculation. *Appl. Microbiol. Biotechnol.* 97, 8859-8873. DOI:10.1007/s00253-013-5228-8.

41. Saranya K. & Kumutha K. (2011). Standardization of the substrate material for large scale production of arbusacular mycorrhizal inoculum. *Int. J. Agr. Sci.* 3, 71-77.

42. Coelho I. R., Pedone-Bonfim M. V. L., Silva F. S. B., Maia L. C. (2014). Optimization of the production of mycorrhizal inoculum on substrate with organic fertilizer. *Brazilian J. Microbiol.* 45, 4, 1173-1178.

43. Posada R. H., Franco L. A., Ramos C., Plazas L. S. , Suàrez J.C., Alvarez F. (2008). Effect of physical, chemical and environmental characteristics on arbuscular mycorrhizal fungi in *Brachiaria decumbens* (Stapf) pastures. *J. Appl. Microbiol.* 104, 132–140. DOI:10.1111/j.1365-2672.2007.03533.x.

44. Douds D. D. Jr., Nagahashi G., Reider C., Hepperly P. R. (2007) Inoculation with arbuscular mycorrhizal fungi increases the yield of potatoes in a high P soil, biological agriculture & horticulture. *Biol. Agr. Hortic.* 25, 1, 67-78. DOI: 10.1080/01448765.2007.10823209.

45. Silva F. S. B., Yano-Melo A. M., Brandão J. A. C., Maia L. C. (2005). Sporulation of arbusaculat mycorrhizal fungi using Tris-HCl buffer in addition to nutrient solutions. *Brazilian J. Microbiol.* 36, 327-332.

46. Basiru S., Mwanza H. P., Hijri M. (2021). Analysis of arbuscular mycorrhizal fungal inoculant Benchmarks. *Microorganisms* 9, 81-99. https://doi.org/10.3390/microorganisms901008.

47. Oviatt P., Rillig M. C. (2021) Mycorrhizal technologies for an agriculture of the middle. *Plants People Planet* 3, 454–461. DOI: 10.1002/ppp3.10177.

48. Faye A., Stewart Z. P., Ndung'u-Magiroi K., Diouf M., Ndoye I., Diope T., et al. (2020). Testing of commercial inoculants to enhance P uptake and grain yield of promiscuous soybean in Kenya. *Sustainability 12*, 3803-3818. doi:10.3390/su12093803.

Click here to access/download;Figure;Figures-Nadieline et al-April 2024.pdf

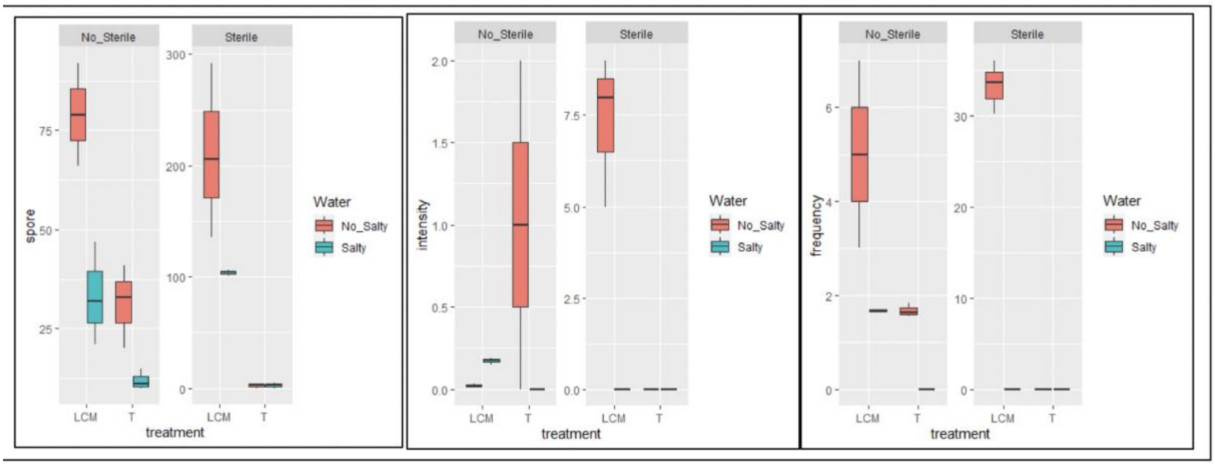

Fig 1. Box plot showing the effect of using salt water and substrate sterilization on inoculum quality parameters (Spore frequency, mycorrhization Intensity and Frequency). *LCM refers to inoculation with the mix of LCM strains; T, non-inoculated; Salty, irrigated with salted water (NaCl, 0.5% W/V); No_Salty, irrigated with unsalted water; Sterile, sterilized peanut shell support; Non_Sterile, unsterilized peanut shell support*.

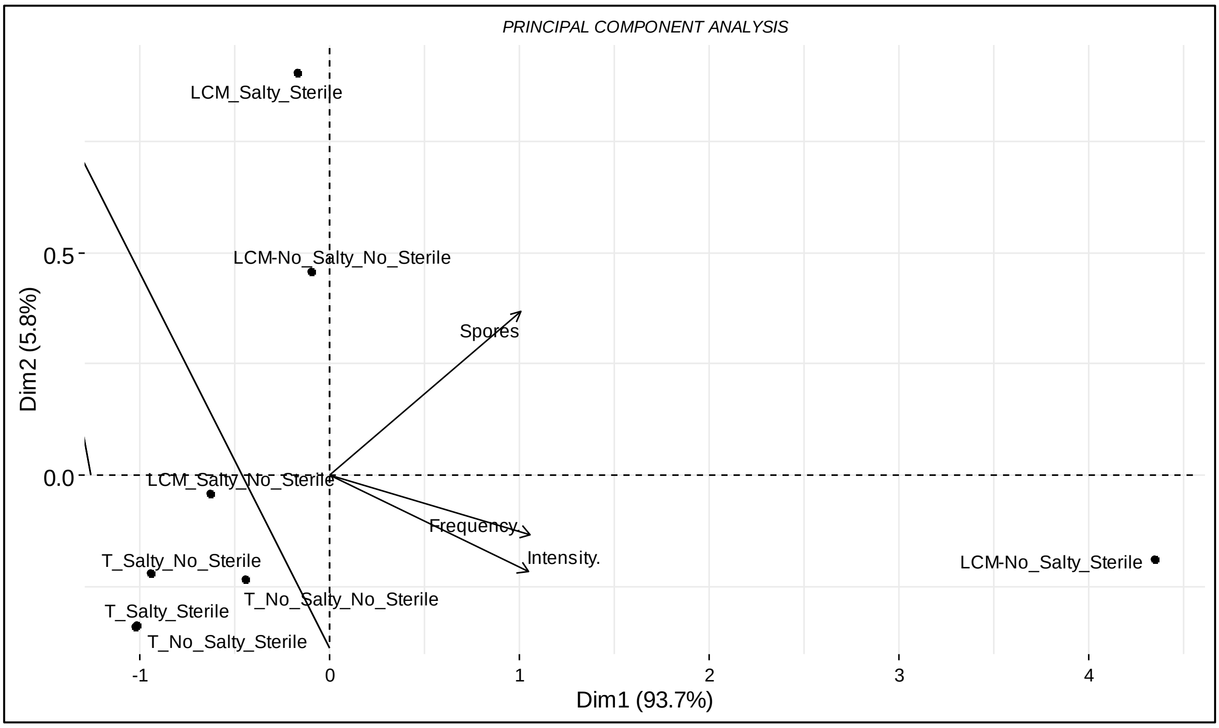

Fig 2. Principal Component Analysis showing the effects of the treatments on the mycorhization using as variables the number of spores, mycorrhization intensity and frequency. *LCM*No Salty*No Sterile: unsterilized support, inoculated and watered with unsalted water; LCM*Salty*No Sterile: unsterilized support, inoculated and watered with salt water; LCM*No Salty*Sterile: sterilized support, inoculated and watered with unsalted water; LCM*Salty*Sterile: sterilized support, inoculated and watered with salt water; T*Salty*Sterile: sterilized support, not inoculated and watered with salt water; T*No Salty*No Sterile: unsterilized support, uninoculated and watered with unsalted water; T*No Salty*Sterile: sterilized support, not inoculated and watered with non-salty water and T*Salty*No Sterile: unsterilized support, uninoculated and watered with salt water.*

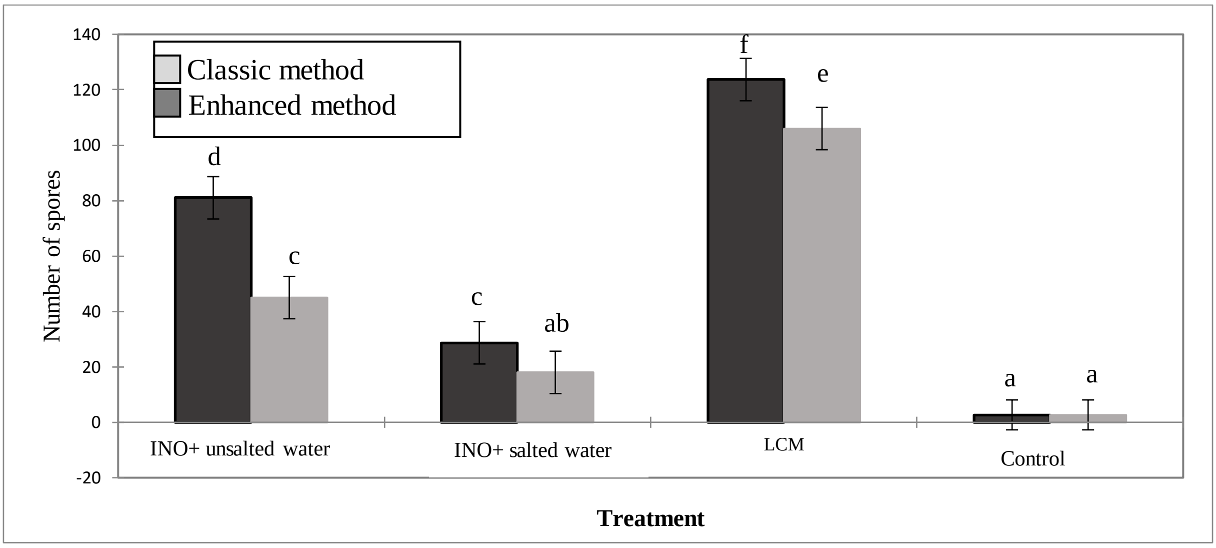

Fig 3. Mean number of spores obtained by classical and improved extraction methods when using peanut shells as growth substrate. *The means of three repetitions per treatment followed by different letters differ according to the Fisher test (p = 0.05). Bars represent standard deviations from the mean (n=3). The comparison is made within each treatment. INO, inoculum originated from the Darou Mousty pilot site obtained with salted and unsalted irrigation water; LCM, inoculum obtained from starter of Joint Microbiology Laboratory (LCM) on peanut shells in the laboratory; control, uninoculated corn grown in peanut shell*.
