# Supplementary material for: Development of on-farm AMF inoculum production for sustainable agriculture in Senegal
Source: PLoS One. 2024 Nov 27;19(11):e0310065. doi: 10.1371/journal.pone.0310065 (PMC11602082; doi:10.1371/journal.pone.0310065)
Supplement: Attachment — Submitted filename: Responses to Reviewers.pdf [file pone.0310065.s008.pdf]

Dear Editor,

The Authors would like to thank the editorial team as well as reviewers for their careful and constructive comments. We have been through Journal Requirements and Reviewers concerns and made revisions accordingly. The revised manuscript submitted integrates all changes made and we have responded to each point below using green font to facilitate the reading.

1. When submitting your revision, we need you to address these additional requirements.

Please ensure that your manuscript meets PLOS ONE's style requirements, including those for file naming. The PLOS ONE style templates can be found at https://journals.plos.org/plosone/s/file?id=wjVg/PLOSOne_formatting_sample_main_body.pdf and https://journals.plos.org/plosone/s/file?id=ba62/PLOSOne_formatting_sample_title_authors_affiliations.pdf.

Response to Editor point 1:

The manuscript has been reviewed and formatted following PLOS ONE's requirements.

2. We note that the grant information you provided in the 'Funding Information' and 'Financial Disclosure' sections do not match.

When you resubmit, please ensure that you provide the correct grant numbers for the awards you received for your study in the 'Funding Information' section.

3. Thank you for stating the following financial disclosure:
 [OAPI funding 27714 and IRD funding MySen].
Please state what role the funders took in the study. If the funders had no role, please state: ""The funders had no role in study design, data collection and analysis, decision to publish, or preparation of the manuscript.""
If this statement is not correct you must amend it as needed.
Please include this amended Role of Funder statement in your cover letter; we will change the online submission form on your behalf.

4. Thank you for stating the following in the Acknowledgments Section of your manuscript:
[This work was supported by the African Intellectual Property Organization (OAPI) funding 27714 and the Senegalese Innovative Business Incubator (INNODEV) between 2013 and 2016, and by the French National Research Institute for Sustainable Development (IRD) funding MySen between 2017 and 2019. The authors thank the management of the Joint Microbiology Laboratory IRD-ISRA-UCAD (LCM) for supporting and the authorities of the commune of Darou Mousty and the farmers for the facilities granted to this work.]
We note that you have provided funding information that is not currently declared in your Funding Statement. However, funding information should not appear in the Acknowledgments section or other areas of your manuscript. We will only publish funding information present in the Funding Statement section of the online submission form.

Please remove any funding-related text from the manuscript and let us know how you would like to update your Funding Statement. Currently, your Funding Statement reads as follows:
 [OAPI funding 27714 and IRD funding MySen]

Please include your amended statements within your cover letter; we will change the online submission form on your behalf.

Thank for these clarifications, regarding points 2, 3 and 4, we have now removed any funding related text from the manuscript and sections "Funding information" and "Financial Disclosure" have been corrected and submitted online as follow:

Funding information: "This work was supported by the African Intellectual Property Organization (OAPI) funding 27714 received in 2014 and by the French National Research Institute for Sustainable Development (IRD) funding Coup de pouce "MySen" received in 2017.

Financial Disclosure: "The funders had no role in study design, data collection and analysis, decision to publish, or preparation of the manuscript."

5. We note that your Data Availability Statement is currently as follows: [All relevant data are within the manuscript and its Supporting Information files]

Please confirm at this time whether or not your submission contains all raw data required to replicate the results of your study. Authors must share the "minimal data set" for their submission. PLOS defines the minimal data set to consist of the data required to replicate all study findings reported in the article, as well as related metadata and methods (https://journals.plos.org/plosone/s/data-availability#loc-minimal-data-set-definition).

For example, authors should submit the following data:

- The values behind the means, standard deviations and other measures reported;
- The values used to build graphs;
- The points extracted from images for analysis.

Authors do not need to submit their entire data set if only a portion of the data was used in the reported study.

If your submission does not contain these data, please either upload them as Supporting Information files or deposit them to a stable, public repository and provide us with the relevant URLs, DOIs, or accession numbers. For a list of recommended repositories, please see https://journals.plos.org/plosone/s/recommended-repositories.

If there are ethical or legal restrictions on sharing a de-identified data set, please explain them in detail (e.g., data contain potentially sensitive information, data are owned by a third-party organization, etc.) and who has imposed them (e.g., an ethics committee). Please also provide contact information for a data access committee, ethics committee, or other institutional body to which data requests may be sent. If data are owned by a third party, please indicate how others may request data access.

Response to Editor point 5: please accept our apologies for not including these data in the first version of the manuscript. We have now included as Supporting information all raw data obtained as new dataset files for each one of the tables and figures presented in this work.

Supporting information related to raw data linked to the submitted work:

S1 dataset: raw data refereeing to Table 1 "Mycorrhization parameters (mean data) of AMF starters used for inoculum production on the pilot site"

S2 dataset: raw data refereeing to Table 2 "Characteristics of the inoculum produced on agricultural residues alone or mixed with sand in Leonard jars"

S3 dataset: raw data refereeing to Table 3 "Characteristics of the inoculum produced on agricultural residues in pots"

S4 dataset: raw data refereeing to Table 4 "Characteristics of the inoculum produced on the peanut shell at the pilot site"

S2 and S3 Fig dataset: raw data refereeing to Fig 2 “Box plot showing the effect of using salt water and substrate sterilization on inoculum quality“ and Fig 3 “Principal Component Analysis showing the effects of the treatments on the mycorhization using as variables the number of spores, mycorrhization intensity and frequency“.

S4 Fig dataset: raw data refereeing to Fig 4 “Mean number of spores obtained by classical and improved extraction methods when using peanut shells as growth substrate“.

6. We note that you have included the phrase “data not shown” in your manuscript. Unfortunately, this does not meet our data sharing requirements. PLOS does not permit references to inaccessible data. We require that authors provide all relevant data within the paper, Supporting Information files, or in an acceptable, public repository. Please add a citation to support this phrase or upload the data that corresponds with these findings to a stable repository (such as Figshare or Dryad) and provide and URLs, DOIs, or accession numbers that may be used to access these data. Or, if the data are not a core part of the research being presented in your study, we ask that you remove the phrase that refers to these data.

Response to Editor point 6: we have removed this part of the sentence as it is not dispensable and was not appropriate as sand sterilization was not used in the present study.

The new sentence is now:” The shells were moistened with tap water and sterilized by cooking for 30 minutes in metal barrels heated with firewood by monitoring the cooking and avoiding burning (S1 Fig.).

Review Comments to the Author

Please use the space provided to explain your answers to the questions above. You may also include additional comments for the author, including concerns about dual publication, research ethics, or publication ethics. (Please upload your review as an attachment if it exceeds 20,000 characters)

Reviewer #1: Thank you for the opportunity to review for the esteemed journal, PLOS ONE.

Regarding the manuscript, I regret to say that it may not align with the interests of your journal's readership. Major revisions are necessary before considering it for publication in the PLOS ONE. Nonetheless, I recommend the authors to undertake substantial revisions to improve the manuscript.

We thank Reviewer 1 for his recommendations. We believe that the proposed study involving multiple actors is of wide interest and fits to PLOS ONE aims. We have modified the text so as to clarify all sections as recommended by reviewer 1, please see below for point by point responses to reviewer 1 comments and associated revisions made in the main text.

It is advisable to include some information about materials and methods in the abstract.

> Additional information about materials and methods has been included in the abstract.

The referencing style should adhere to the journal's guidelines.

>The referencing style has been revised.

The abstract is not presented in a manner that the readers understand the main results of this study.

> We have added the following text in the Abstract section regarding materials and methods: “Peanut shell, rice husk, sugar cane bagasse and millet ears were tested in Leonard jars and pots as alternatives to conventional sand production substrate for the multiplication of mycorrhizal fungi *Glomus aggregatum* IR27, *Funneliformis mosseae*, *Rhizophagus irregulares* and *Glomus fasciculatum* R10.**”**

and main results of the study: **“**Significant results were obtained on the peanut shell. Under mass production conditions in farm scale, *Glomus aggregatum* IR27 showed the best mycorization characteristics with 19.76% intensity and 88.93% frequencies. The study highlighted the critical considerations of irrigation water salt content and substrate sterilization as essential parameters to ensure optimal development of mycorrhizal propagules. Water containing 0.5% salt inhibited the mycorrhization. This negative effect of salt was much more accentuated in unsterilized peanut shell substrate than in sterilized one**”**

The Materials and Methods section requires additional details about experimental conditions.

There is a lack of comprehensive description regarding experimental design, materials, and methods.

> Details about materials and methods for the production of in farm AMF production have been described as a new figure as supporting information (please see Fig 1).

Please explicitly state whether the objectives are novel.

> We have emphasized the novelty of the objectives to produce on farm AMF by producers using “crop residues…” in the Introduction: “This experimental enterprise constitutes a pioneering initiative, potentially replicable in other agricultural areas. Its sustainability relies on the integration of renewable agricultural residues into the mycorrhizal inoculum production path in order to ensure the availability of the product on a large scale.”

The below updated references would be useful:
https://doi.org/10.1016/B978-0-443-15884-1.00002-6
https://doi.org/10.1016/j.indcrop.2022.115557
https://doi.org/10.1016/j.eja.2021.126283
https://doi.org/10.1111/jam.15326
https://doi.org/10.1016/j.indcrop.2016.06.024
https://doi.org/10.1016/j.eja.2016.01.006

> We have decided to include three references (Seyyed Ali Sadegh Sadat Darakeh et al., 2022, Weria Weisany, 2021 and Loftabadi et al., 2022) that we think will improve the manuscript; we do not think that the other proposed references are essential for the comprehension of the article.

The introduction lacks coherence, with some sentences appearing conceptually disconnected, particularly towards the conclusion. Given the significance of the study, more attention should be devoted to refining the introduction.

Clear articulation of hypotheses and objectives is essential.

> We have revised the introduction, the conclusion connects well with the objectives now.

“This study endeavors to explore the feasibility of producing elite AMF inoculum on a semi-industrial scale within agricultural production sites. The simplicity and efficiency of the technology lays the foundations for the creation of a local sector for the production and distribution of inoculum of endomycorrhizal fungi in Senegal. Ultimately, it contributes to stimulate the integration of symbiotic microorganisms into agricultural practices in the form of bio-stimulants.”

The Materials and Methods section necessitates restructuring for clarity, as some paragraphs are poorly organized, leading to confusion.

> Details about materials and methods for the production of in farm AMF production have been described as a new figure as supporting information (please see S1 Fig).

Insufficient presentation, especially in terms of mean comparison, and inadequate description of results are evident. Additionally, there is an overabundance of poorly annotated illustrations and tables.

> The tables have been transferred to the supporting information and the details have been provided in terms of mean comparison. Additional data was provided in the supporting information.

It is challenging to discern how conclusions were derived from the illustrations in some instances, while other effects were not adequately presented.

>The conclusion section has been revised and more focused on the present study. The prospects for increasing of AMF production units have been identified.

The discussion lacks depth, primarily consisting of a list of references for each measured variable. There is a notable absence of analysis on experimental processes and practical implications. The discussion fails to contextualize the experimental findings or justify the necessity of the study compared to existing literature.

>The Discussion section has been revised and was more contextualized. The effort was made to reconcile the results obtained with data from the literature. The connection was made with the objectives.

The discussion lacks integration among different variables measured in the experiment, failing to draw simultaneous conclusions from their measurements.

> We have taken these suggestions into account and revised all discussion section.

Reviewer #2:

Abstract
- Lion 41-42: I think this information is inaccurate. There is a lot of research conducted in African countries in this regard, and commercial biofertilizer compounds contain mycorrhizal fungi for use in agricultural fields.

> We are observed that industrial inoculum production in Africa is limited to a few countries. Commercial products do not often contain native strains adapted to all environments. They are not accessible to farmers. This study aims to develop a simple protocol for producing quality inoculum whose know-how can be easily transferred to farmers to ensure their autonomy.

Introduction
- Although the introduction section is large, it does not contain specific information about the study factors
- Most of the introduction is general talk that needs to be shortened
- The introduction needs to be strengthened with previous studies that address the study factors

> The Introduction section has been improved: shortened, enriched with bibliographic data and focused on the objectives and originality:
"This experimental enterprise constitutes a pioneering initiative, potentially replicable in other agricultural areas. Its sustainability relies on the integration of renewable agricultural residues into the mycorrhizal inoculum production path in order to ensure the availability of the product on a large scale. This study endeavors to explore the feasibility of producing elite AMF inoculum on a semi-industrial scale within agricultural production sites. The simplicity and efficiency of the technology lays the foundations for the creation of a local sector for the production and distribution of inoculum of endomycorrhizal fungi in Senegal. Ultimately, it contributes to stimulate the integration of symbiotic microorganisms into agricultural practices in the form of bio-stimulants"

Reviewer #3: Manuscript ID: PONE-D-24-13955
Development of on-farm AMF inoculum production for sustainable agriculture in Senegal
Dear editor,

The paper is original. The topic is interesting and may have application potential. The authors aim to investigate the feasibility of producing mycorrhizal inoculum on a semi-industrial farm scale. Agricultural residues were tested as replacements of sand. In the other experiments they have tested the effects of salinity water and soil sterilization on AMF prolifiration and production. The experiments are well designed and the data are more or less enough to interpret the results. The text is written relatively well.

> We thank the reviewer 3 for these positive and encouraging comments.

The results were relatively well-presented and discussed but I have some more concerns as they have been mentioned in the pdf file as note which has been attached.

> We have taken the notes into consideration; the details have been provided in the text.

Line 36: please enhance the results in the Abstract quantitatively. Most of this part is introduction and methodology

> Details were provided on the methodology and results obtained

Line 59: The introduction part is relatively well-written. please mention the reasons t you chose these four fungi species.

> These strains were used for their performances observed in previous work referenced in the document

Line 77: small letters for phosphorus and potassium
> letter size has been corrected

Line 89: is this correct? which plant is this?

> Yes, it's correct. It's *Acacia albida* (Please see Diop et al., 1994)

Line 135 "and"
>correction made

Line 175 : specific reason to chose this plant as host?
>Explanation has been provided in the text:

"Corn (*Zea mays* L.) was chosen as the host plant that is justified by the properties of high mycotrophy and root biomass comprising a large number of fine roots, thus facilitating the establishment of mycorrhizae"

Line 178: the time of 3 weeks seems too long. what about the humidity and temperature and other conditions?

>Three weeks are followed to ensure complete drying of the aerial biomass. Temperature and humidity were not measured in this experiment. Recent studies show that the temperature at depth of the substrate could reach 40°-50°C.

Line 179: why this part of plants were removed?

> the aerial part has been cut to facilitate the extraction of the roots.

Line 184: why the amount of hypha and vesicles were not measured?

>We do not have the technical equipments to carry out these measurements but we agree that it's interesting to measure.

Line 189: Are you sure that you have to boil for an hour?

> It is a mistake. The inculation is done for 30 minutes. The correction has been made in the text

Line 192: please mention it here that how many replications did you tested for each sample?

> clarification has been made

« A total of 100 root fragments were taken randomly for each treatment. On slide contained 20 fragments, with 5 repetitions for each treatment «

Line 196: Did you use both references of Trouvelot and Philips and Hayman to measure?

> No, just the method of Trouvelot at al.

Line 213: Long sentence!

>The sentence has been modified:
"Crushed peanut shells were used as substrate. The shells were moistened with tap water and sterilized by cooking for 30 minutes in metal barrels heated with firewood by monitoring the cooking and avoiding burning (S1_Fig)".

Line 216: At which growing stage of plant did you added the AMF starters? I assume in the same time as you sown...

>Yes, the AMF starters were added in the same time as we sown. The precision has been added to the text.
"In each container, a total of 200 g of microbial starter was deposited at a depth of 2-3 cm at the same time as corn sowing. Each treatment comprised sixteen repetitions".

Line 224: what do you mean when you say you have combined with eleven other ...? why dont you tested each aliquots without mixing?

>We wanted to analyze a randomized sample. In addition, analyzing each repetition presented a large number of samples. We have made the clarification in the text.

"Subsequently, samples of 100 g from each repetition were collected and mixed for each treatment to create a random sample inoculum for quality control at the laboratory in Dakar".

Line 241: Please write it as: Effect of salty water in watering plant or effect of soil salinity....

>Correction has been made.

"Effect of salty water in watering plant and substrate sterilization on inoculum quality"

Line 246: Why four strains have been mixed? how would you know the effect of each strain?

>We used the mixture of strains to avoid the specified infection of corn under these growing conditions in order to ensure infection and be able to move forward with the development of the protocol.

We have made this clarification in the text.

“To guarantee infection and avoid possible infection specificity, starter comprising a mixture of the four strains in equal proportion, was used for this test”.

Line 256: uninoculated

> it's corrected

Line 284: Captions need to be explained more in details. Write in the caption what are substrate ? also ***? and what data have been compared?
These tabled missed some detailes and explanations

> The table has been transferred to the supporting information S2 Table and the details have been provided

correct the word ((substrate))

> it's corrected

Line 287: Same comments as table 2

> The table has been transferred to the supporting information S3 Table and the details have been provided

Table 4: Does it mean that mixing sand with substrate has antagonistic effect?

>No, we don't think it's an antagonistic effect. It could be a reduction in nutrients.

Line 347: Put a dot
> it's done

Table 8: write here or in the caption, what is substrate here. Also indicate the meaning of *** and ** and*

what is this ?

> The table has been transferred to the supporting information S4 Table and the details have been provided. We corrected “eau” by water.

Line 450: Write scientific names in italic

> it's done

References: need minor corrections
>Corrections have been made on the journal format
