# Supplementary material for: Development of on-farm AMF inoculum production for sustainable agriculture in Senegal
Source: PLoS One. 2024 Nov 27;19(11):e0310065. doi: 10.1371/journal.pone.0310065 (PMC11602082; doi:10.1371/journal.pone.0310065)
Supplement: Attachment — Submitted filename: Responses to Reviewers.pdf [file pone.0310065.s009.pdf]

Dear Editor,

The Authors would like to thank the editorial team as well as reviewers for their constructive comments. We have made revisions accordingly the reviewers' comments. The revised manuscript submitted integrates all changes made and we have responded to each point below using green font to facilitate the reading.

Journal Requirements:

Please review your reference list to ensure that it is complete and correct. If you have cited papers that have been retracted, please include the rationale for doing so in the manuscript text, or remove these references and replace them with relevant current references. Any changes to the reference list should be mentioned in the rebuttal letter that accompanies your revised manuscript. If you need to cite a retracted article, indicate the article's retracted status in the References list and also include a citation and full reference for the retraction notice.

>The reference list has been revised. No reference has been retracted. Four new references were cited as below.

Review Comments to the Author

6. Review Comments to the Author

Reviewer #1: The authors revised the entire manuscript according to the recommendations of the reviewers and the editor. It has been significantly improved, and the discussion is now sufficiently critical and can therefore be considered for acceptance.

>We thank Reviewer 1 for his positive appreciations.

Reviewer #2: The introduction has been greatly shortened. Please add some research to the introduction that highlight the importance of mycorrhiza on plant production, especially in arid and semi-arid regions such as Africa. For example: https://doi.org/10.1016/j.rhisph.2024.100852

>We thank Reviewer 2 for his positive appreciations. Additional researches have been mentioned in the introduction that we think will improve the manuscript;

"Additionally, it was demonstrated across diverse agro-ecological zones adaptability to saline and water stress conditions [19] and improving of plant production in arid and semi-arid zones [20, 21, 22, 23]"

The references' list has been updated:

20. Shalaby OA, Ramadan MEl-SR. Mycorrhizal colonization and calcium spraying modulate physiological and antioxidant responses to improve pepper growth and yield under salinity stress. Rhizosphere. 2024;29,100852. https://doi.org/10.1016/j.rhisph.2024.100852.

21. Ould Amer S, Aliat T, Kucher DE, Bensaci OA, Rebouh NY. Investigating the potential of arbuscular mycorrhizal fungi in mitigating water deficit effects on durum wheat (*Triticum durum* Desf.). Agriculture. 2023;13(3):552-68. https://doi.org/10.3390/agriculture13030552.
22. Duan H-X, Luo C-L, Li J-Y, Wang B-Z, Naseer M, Xiong Y-C. Improvement of wheat productivity and soil quality by arbuscular mycorrhizal fungi is density- and moisture-dependent. Agron Sustain Develop. 2021;41:3-12. https://doi.org/10.1007/s13593-020-00659-8.
23. Wahab A, Muhammad M, Munir A, Abdi G, Zaman W, Ayaz A et al. Role of arbuscular mycorrhizal fungi in regulating growth, enhancing productivity, and potentially influencing ecosystems under abiotic and biotic Stresses. Plants. 2023;12:3102-40. https://doi.org/10.3390/plants12173102.
